# Design, Synthesis, and Biological Evaluation of Desmuramyl Dipeptides Modified by Adamantyl-1,2,3-triazole

**DOI:** 10.3390/molecules26216352

**Published:** 2021-10-21

**Authors:** Vesna Petrović Peroković, Željka Car, Josip Draženović, Ranko Stojković, Lidija Milković, Mariastefania Antica, Đani Škalamera, Srđanka Tomić, Rosana Ribić

**Affiliations:** 1 Department of Chemistry, Faculty of Science, University Zagreb, Horvatovac 102, HR-10000 Zagreb, Croatia; vpetrovi@chem.pmf.hr (V.P.P.); zcar@chem.pmf.hr (Ž.C.); josip.drazenovic@irb.hr (J.D.); dskalamera@chem.pmf.hr (Đ.Š.); stomic@chem.pmf.hr (S.T.); 2 Division of Molecular Medicine and Division of Molecular Biology, Ruđer Bošković Institute, Bijenička cesta 54, HR-10000 Zagreb, Croatia; stojkov@irb.hr (R.S.); lidija.milkovic@irb.hr (L.M.); mariastefania.antica@irb.hr (M.A.); 3 Department of Nursing, University Center Varaždin, University North, Jurja Križanića 31b, HR-42000 Varaždin, Croatia

**Keywords:** mannose, desmuramyl peptide, adamantyl-triazole, immunostimulant activity

## Abstract

Muramyl dipeptide (MDP) is the smallest peptidoglycan fragment able to trigger the immune response. Structural modification of MDP can lead to the preparation of analogs with improved immunostimulant properties, including desmuramyl peptides (DMPs). The aim of this work was to prepare the desmuramyl peptide (L-Ala-D-Glu)-containing adamantyl-triazole moiety and its mannosylated derivative in order to study their immunomodulatory activities in vivo. The adjuvant activity of the prepared compounds was evaluated in a murine model using ovalbumin as an antigen, and compared to the reference adjuvant ManAdDMP. The results showed that the introduction of the lipophilic adamantyl-triazole moiety at the C-terminus of L-Ala-D-Glu contributes to the immunostimulant activity of DMP, and that mannosylation of DMP modified with adamantyl-triazole causes the amplification of its immunostimulant activity.

## 1. Introduction

**Figure 1:**
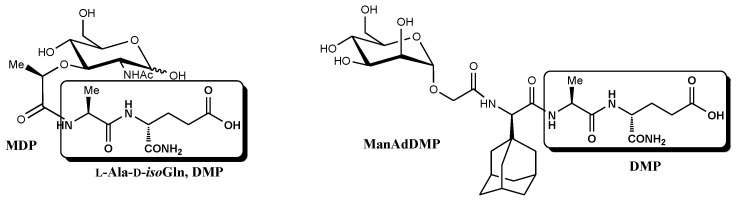
Structure of MDP, adamantane-containing DMP, and its mannose derivative.

Peptidoglycans are conserved polymeric constituents of Gram-positive and Gram-negative bacteria that act as agonists of pathogen-recognition receptors (PRRs) and, therefore, stimulate the immune response [1,2,3]. PRRs in general are classified into Toll-like receptors (TLRs), RIG-I-like receptors (RLRs), NOD-like receptors (NLRs), and C-type lectin receptors (CLRs) [4]. Muramyl dipeptide (MDP, *N*-acetylmuramyl-L-alanyl-D-*iso*glutamine) is the smallest peptidoglycan fragment (Figure 1) able to trigger the immune response via activation of the nucleotide-binding oligomerization domain-containing protein 2 (NOD2). Structural details regarding the activation of NOD2 are still not fully understood but, based on the crystal structure of the rabbit NOD2 in the ADP-bound form, an MDP binding site is proposed [5].

Structure–activity studies of muropeptides have been performed in order to prepare an adjuvant with improved properties of the MDP molecule, and lipophilic analogs and derivatives of MDP have shown improved immunostimulant activity [6,7,8,9].

Up to now, our research has been directed towards MDP analogs lacking the *N*-acetylmuramyl group, called desmuramyl peptides (DMPs). We have prepared DMPs containing lipophilic adamantane moieties attached at the *N*- and *C*-termini of L-Ala or D-*iso*Gln, as well as their mannosylated derivatives. The results indicate that mannosylation of DMPs contributes to the stimulation of the immune response [10,11], possibly by affecting the immune response via the mannose receptor family [12,13]. The immunomodulatory activities of all prepared DMPs and their mannosylated derivatives were estimated in vivo, in murine models, based on the secondary humoral response to ovalbumin as a test antigen. In structure–activity relationship (SAR) studies, we have shown that glycoconjugates that contain a glycolyl linker between mannose and the DMP moiety exhibit a stronger adjuvant activity than analogs containing a hydroxyisobutyryl linker [11]. The most active adjuvant in this class of compounds is ManAdDMP (Figure 1), which has adamantane in the form of adamantylglycine between mannose and L-Ala. We have confirmed that MDP and ManAdDMP with a free carboxyl group at the side chain of the *iso*Gln form strong interactions with the NOD2 receptor. Specifically, free γ-COOH makes the greatest contribution to the NOD2 binding. Furthermore, a contribution of hydrophilic mannose and lipophilic adamantane to the NOD2 binding was also observed [14].

Therefore, within this study we synthesized DMP derivatives with the adamantane substituent attached to the C-terminus of the dipeptide, at α-COOH of D-Glu. Furthermore, herein we describe a strategy for the preparation of an adamantyl-triazole structure, which was then coupled with the dipeptide part. The immunomodulatory properties of the prepared adamantyl-triazole DMP and its mannosylated derivative were estimated in vivo in a well-established murine model [8,10,11], and compared with the reference ManAdDMP adjuvant.

## 2. Results

### 2.1. Chemistry

In this work, we describe the preparation and characterization of adamantyl-triazole DMP **8** and its mannose derivative **14**. The key step in the synthesis of compound **8** was the conjugation of L-alanyl-D-*iso*glutamine and adamantyl-triazole **3**.

**Scheme 1:**
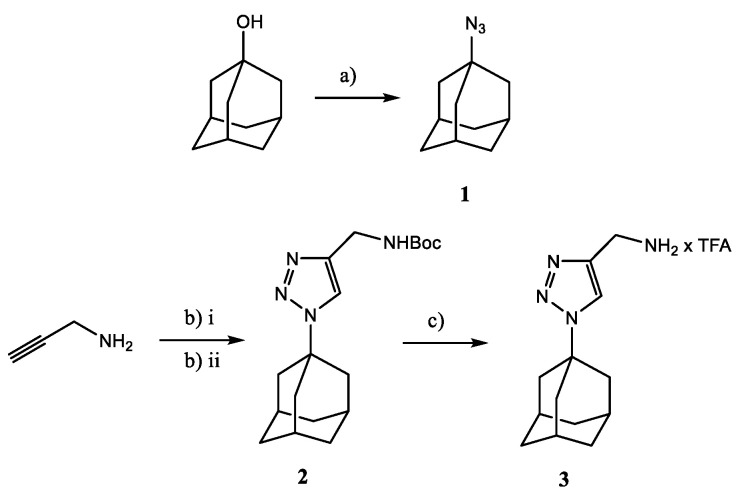
Reagents and conditions: (a) NaN_3_, H_2_SO_4_, CHCl_3_, rt, 3 h, 87%; (b) (i) Boc_2_O, TEA, 1,4-dioxane, H_2_O, 0 °C to rt, 90 min, (ii) **1**, Na ascorbate (aq), CuSO_4_ × 5 H_2_O (aq), Ar, 35 °C, 24 h, 97%; (c) TFA, dry CH_2_Cl_2_, rt, 2 h, 87%.

Compound **3** was prepared as shown in Scheme 1, starting from commercially available 1-adamantanol using a known procedure [15]. A modified one-pot method [16] was used for the preparation of Boc-protected derivative **2,** which was subsequently deprotected using TFA, with good yields.

**Scheme 2:**
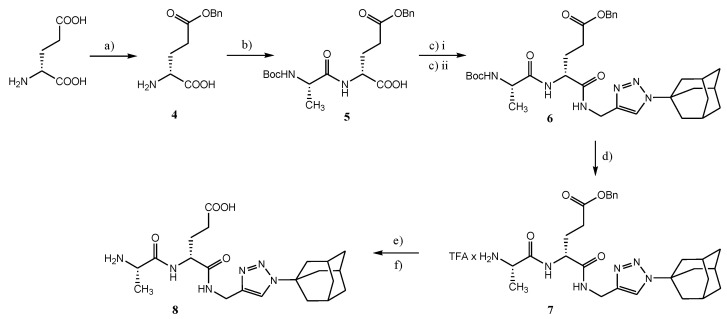
Reagents and conditions: (a) BnOH, BF_3_ × Et_2_O, TEA, THF, Ar, rt, 20 h, 61%; (b) Boc-L-AlaOSu, TEA, THF, Ar (g), 0 °C to rt, 24 h, 95%; (c) (i) **3**, *N*-methylmorpholine, *n*-butyl chloroformate, THF, Ar (g), −10°C, 2 h, (ii) NaHCO_3_ (aq), rt, 1 h, 95 %; (d) TFA, dry CH_2_Cl_2_, rt, 4 h, 81%; (e) H_2_, 10% Pd/C, 96% EtOH, 2.8 bar, rt, 4 h, 85%; (f) HCOONH_4_ (aq).

Peptide building blocks were prepared starting from unprotected D-Glu (Scheme 2). In the first step, according to the procedure previously described [17], the carboxyl group of the side chain of D-Glu was selectively protected to produce compound **4** (Figure S3) with a good yield. Compound **4** was then coupled with commercially available Boc-L-Ala-OSu in the presence of triethylamine (TEA) to form dipeptide **5** (Figure S4). The *n*-butyl chloroformate/*N*-methylmorpholine coupling method was used for the conjugation of **5** with adamantyl-triazole derivative **3**. Thus, compound **6** was prepared with an excellent yield. Boc and benzyl protection were removed using standard and efficient procedures, and compounds **7** and **8**, respectively, were obtained.

**Scheme 3:**
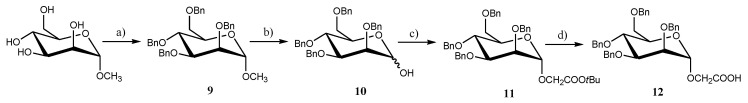
Reagents and conditions: (a) KOH, BnBr, 100 °C, dry 1,4-dioxane, 24 h, 61%; (b) HOAc, H_2_SO_4_, 90 °C, 5.5 h, 30%; (c) BrCH_2_COO*t*-Bu, K_2_CO_3_, dry DMF, 48 h, 62%; (d) TFA, dry CH_2_Cl_2_, rt, 24 h, 76%.

The preparation of mannose precursor **12** is shown in Scheme 3. Benzylated mannopyranose **10** was prepared using a slightly modified version of the procedure described by Koto et al. [18]. S_N_2 substitution of bromine from *tert*-butyl bromoacetate with 2,3,4,6-tetra-*O*-benzyl-α-D-mannopyranose **10** in the presence of potassium carbonate produced compound **11**. The *tert*-butyl group of the resulting compound **11** was removed using trifluoroacetic acid (TFA), and the benzylated mannoside **12** with a free carboxyl group was obtained for coupling with the peptide precursor (Scheme 3).

**Scheme 4:**
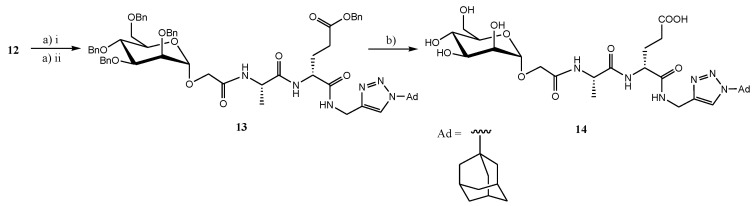
Reagents and conditions: (a) (i) EDC × HCl, HOBt, dry DCM, 0 °C, (ii) **7**, TEA, dry 1,4-dioxane, 0 °C to rt, 24 h, 95%; (b) H_2_, 10% Pd/C, 96% EtOH, 2.8 bar, rt, 48 h, 35%.

The synthesis of mannose conjugate **13** was performed using the standard EDC/HOBt coupling method with triethylamine as a base, with an excellent yield (Scheme 4). Debenzylation of **13** via catalytic hydrogenolysis produced the final product **14**, unequivocally identified by HPLC analysis, ^1^H NMR and ^13^C NMR spectroscopies, and mass spectrometry.

### 2.2. Evaluation of Immunostimulant Activity

The adjuvant activity was estimated by the immunostimulant effect on the secondary humoral response of a well-established model antigen ovalbumin (OVA) in BALB/c mice, according to previously described in vivo studies [11,19]. Overall anti-OVA IgG, and subclasses of IgG (anti-OVA IgG1 and anti-OVA IgG2a) as indicators of Th1 and Th2 of the immune response, were measured in the murine sera after a second booster. The adjuvant activity of the newly synthesized compounds was compared to the reference ManAdDMP, which is able to switch the immune response towards a pronounced Th2-type immune reaction, as was described for MDP [11].

**Figure 2:**
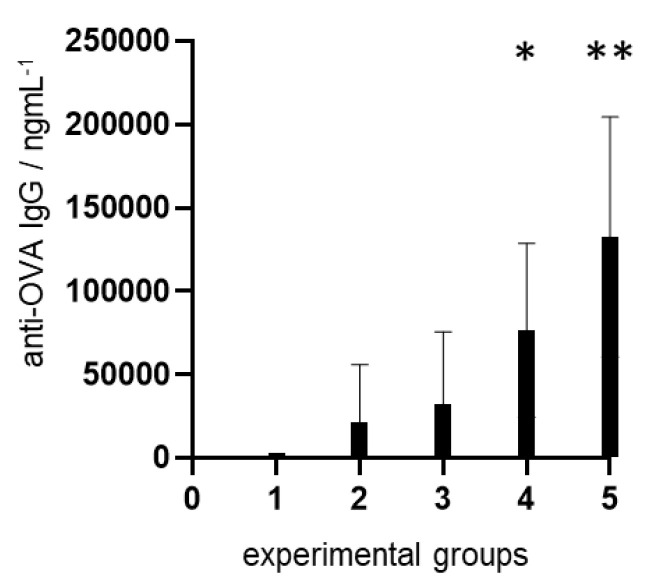
The effect of synthesized DMPs on the production of anti-OVA IgG in BALB/c mice immunized with OVA as an antigen. Experimental groups: 1—control group; 2—OVA alone; 3—OVA + ManAdDMP; 4—OVA + **8**; 5—OVA + **14**. Bar graphs represent average values from individual mice from each group (*n* = 5). * *p* < 0.05 and ** *p* < 0.01 denote statistical significance in comparison to the control group.

In general, when compared to the group treated with no adjuvant (OVA alone), an enhancement in total anti-OVA IgG antibody production was observed in all groups (Figure 2). High levels of IgG antibody were present even in the OVA-treated group, leading to relatively weak stimulation of total antibody production in the ManAdDMP-injected group. Compound **8** elicited a stronger immune response than OVA alone or the ManAdDMP-injected group. The enhancement in total anti-OVA IgG antibody production by adamantyl-triazole derivative **8** was statistically significant (*p* < 0.05). Immunization with compound **14,** which has compound **8** attached to mannose through the glycolyl linker, led to the highest and most statistically significant increase in the specific IgG response (*p* < 0.01), in comparison to the control group. These results lead to the conclusion that α-COOH is a suitable position for the attachment of adamantane in this class of compounds.

**Figure 3:**
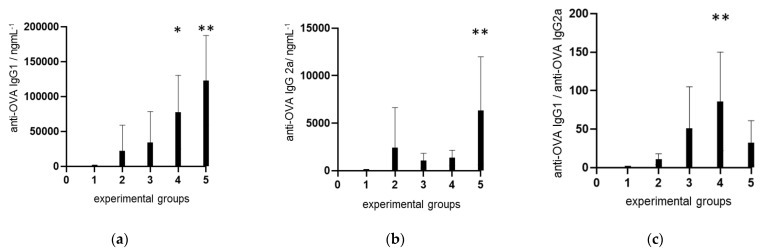
The effect of synthesized DMPs on the production of anti-OVA IgG subtypes: anti-OVA IgG1 (**a**), anti-OVA IgG2a (**b**), and IgG1/IgG2a ratio (**c**), in BALB/c mice immunized with OVA as an antigen. Experimental groups: 1—control group; 2—OVA alone; 3—OVA + ManAdDMP; 4—OVA + **8**; 5—OVA + **14**. Bar graphs represent average values from individual mice from each group (*n* = 5). * *p* < 0.05 and ** *p* < 0.01 denote statistical significance in comparison to the control group.

Since it is known that immune adjuvants can enhance or modulate the Th1/Th2 bias of the induced immune response, the isotype profiles of the antigen-specific anti-OVA IgG antibodies IgG1 and IgG2a were quantitatively determined (Figure 3). Specifically, the type of the generated immune response can be indirectly estimated via the quantification of OVA-specific IgG1 (for activation of Th2 type) and IgG2a (for activation of Th1 type), and calculation of the relevant IgG1/IgG2a ratio. When the amount of anti-OVA IgG1 antibodies was measured (Figure 3a), it was observed in all groups that the trend of IgG1 antibody production was similar to those of overall anti-OVA IgG. The highest response, which was also statistically significant, was elicited by compound **8** (*p* < 0.05) and its mannosylated derivative **14** (*p* < 0.01). A statistically significant enhancement of anti-OVA IgG2a production (Figure 3b) was observed only in the group immunized with mannosylated derivative **14** (*p* < 0.01). The type of immune response was indirectly determined by the calculation of the IgG1/IgG2a ratio (Figure 3c). From the IgG1/IgG2a ratio, it was evident that all groups treated with the tested adjuvants had higher values than the group treated with OVA alone, indicating a slight shift toward a more pronounced Th2-type immune response. Compound **8** significantly (*p* < 0.01) switched the immune response towards a pronounced Th2 type, due to the predominance of IgG1 antibodies.

## 3. Discussion

Natural polymeric peptidoglycans—as well as smaller peptidoglycan fragments, such as MDP and its synthetic derivatives—exhibit immunomodulatory properties. To date, a number of structure–activity studies have been reported, and they have included synthetic modifications at the saccharide and peptide parts of the MDP [7,20,21]. Several synthetically modified MDPs have already been used in clinical settings. Furthermore, formulations of delivery systems—for example, liposomes—of these compounds have been found to be of interest. Mifamurtide is a clinically used therapeutic obtained by linking MDP and dipalmitoylphosphatidylethanolamine, because the phospholipid facilitates the incorporation of the peptide into liposomes [22]. Therefore, modifications with lipophilic substituents have been the mainstream of further research. Our research is focused on the adamantane substituent, because adamantane can act as a membrane anchor, and can enable the preparation of systems for targeted drug delivery [23]; it can also be incorporated into a β-cyclodextrin cavity—a powerful supramolecular nanoparticle carrier for targeted drug delivery [24].

As already mentioned in our previous research, we have demonstrated that the introduction of adamantane at γ-COOH significantly reduces the adjuvant activity by disabling key interactions with the NOD2 receptor [14]. Therefore, within this study, we have explored the modification of the D-Glu at the α-COOH group. In addition to better ligand binding to NOD2, the proposed design will also enable greater exposure of the adamantane structure for possible membrane anchoring, compared to those of ManAdDMP analogs [25,26].

Adamantane was connected to the L-Ala-D-Glu pharmacophore over a 1,2,3-triazole moiety, which was obtained via CuI-catalyzed azide/alkyne cycloaddition (CuAAC) of 1-azidoadamanatane and simple propargylamine. The obtained adamantyl-triazole was then coupled to desmuramyl dipeptide using a standard procedure. The developed synthetic methodology, which includes potent CuAAC click chemistry, could be useful for further structural modification of DMPs (the azido component could be coupled with a wide range of structurally diverse alkynes).

The immunomodulatory properties of DMPs were explored in the BALB/c mouse model in two aspects: one aspect was the analysis of the overall production of specific anti-OVA IgG, and the other was the study of Th1- and Th2-type immune response bias. The results show that the adamantyl-triazole DMP derivative induces higher production of anti-OVA IgG than the reference compound, ManAdDMP, which was the most active adjuvant in this class of compounds. This indicates that the introduction of an adamantyl-triazole moiety at α-COOH is preferred for the amplification of immunostimulant activity, while MDP derivatives with a substituted triazole moiety at the C4 position of MurNAc showed lower affinity toward NOD2 [21]. Based on the IgG1/IgG2a ratio (Figure 3c), it is evident that compound **8** shifts the reaction toward a pronounced Th2-type immune response (*p* < 0.01). MDP dominantly induces IgG1 antibody production, and also stimulates Th2-polarized immune response [19,27].

Furthermore, it is well known that muropeptides act as NOD2 agonists, and can act synergistically. For example, NOD2 ligands can augment the adjuvant activity of TLR ligands, and can modulate innate and adaptive immune responses via NLR/TLR crosstalk [3,28,29,30]. The application of multi-PRR activation, in general, represents a prospective approach in the design of vaccines [31]. Mannose receptors are soluble and transmembrane receptors in the CLR family [4]; they initiate an innate immune response, and also activate the acquired immunity. Therefore, mannose ligand binding could also affect the immune response. For this reason, adamantyl-triazole DMP was further modified by mannose, and glycopeptide **14** was prepared by coupling a corresponding DMP **7** and α-*O*-mannoside **12** functionalized by a carboxylic group, followed by debenzylation.

Mannosylation of peptide **8** amplified the production of anti-OVA IgG antibodies. Anti-OVA IgG levels reflect the ability of a potential adjuvant to increase the humoral immune response. Analysis of IgG1 and IgG2a showed that **14** reduced the switch toward the Th2 direction in comparison to parent compound **8**. These results indicate that mannosylation can enhance the production of overall anti-OVA specific antibodies, but can also influence the polarization of the immune response. The new mannosylated DMP **14** with incorporated adamantyl-triazole will therefore be further explored in order to obtain a better insight into a possible PRR crosstalk. Additionally, it will be used for the preparation of liposome formulations in order to additionally affect the polarization of the immune response [32].

## 4. Materials and Methods

### 4.1. Materials

D-Glutamic acid (D-Glu) was obtained from Bachem (Bubendorf, Switzerland), and other reagents and solvents for the synthesis of compounds were obtained from Sigma-Aldrich Corp. (Darmstadt, Germany). Organic solvents were further purified and/or dried using standard methods. Thin-layer chromatography (TLC) was performed on Fluka silica gel (60 F254) plates (0.25 mm). Visualization was achieved using UV light at 254 nm, 10% sulfuric acid, and ninhydrine. Column chromatography was performed on Merck silica gel 60 (size 70–230 mesh ASTM). The purity of compounds was analyzed via HPLC (Shimadzu HPLC) with a photodiode array detector (SPD-M20A) and autosampler (SIL-20ACTH). All tested compounds had purity over 95% at 214 nm. Analyses were conducted on a Shim-pack GIST C-18 column, 250 mm × 4.6 mm, 5 µm, with a flow rate of 1.0 mL/min, at room temperature. The gradient solvent system used was composed of acetonitrile and water. The percentage of acetonitrile at 0, 5, and 8 min was 10, 30, and 10%, respectively. Acetonitrile and water were of HPLC quality. Mass spectra were recorded using an Agilent 6410 MS instrument. The ^1^H and ^13^C NMR spectra of all precursors were recorded using a Bruker AV-III HD spectrometer at 400 MHz (^1^H) and 100 MHz (^13^C). All NMR experiments were performed at 298 K. Chemical shifts were referenced with respect to tetramethylsilane (TMS).

### 4.2. Synthesis

#### 4.2.1. *tert*-Butyl-((1-(adamantan-1-yl)-1*H*-1,2,3-triazol-4-yl)-methyl)-carbamate (**2**)

Freshly distilled propargylamine (125 μL, 1.95 mmol) was dissolved in a mixture of 1,4-dioxane and water (3.5 mL, 1,4-dioxane/water, 4:3). The mixture was cooled to 0 °C, and then Boc_2_O (608 mg, 2.79 mmol) and TEA (405 μL, 2.91 mmol) were added. After 90 min of mixing at room temperature, 1-azidoadamantane (315 mg, 1.78 mmol), 1M aqueous solution of sodium l-ascorbate (798 μL), and 1M solution of copper(II) sulfate pentahydrate (266 μL) were added to the reaction mixture. The reaction mixture was heated to 35 °C in an argon atmosphere. The reaction was monitored via TLC (DCM/EtOAc, 3:1). After 24 h, the solvent was removed in vacuo. The residue was suspended in water and extracted with EtOAc. The organic layer was dried over anhydrous sodium sulfate. After filtration, the solvent was removed in vacuo. The product was purified via column chromatography on silica gel (DCM/EtOAc, 20:1 to 1:1), and 547.5 mg (97%) of compound **2** was obtained as a white solid. *R*_f_ = 0.31 (DCM/EtOAc, 3:1). ^1^H NMR (CDCl_3_) *δ* 7.56 (s, 1H, CH, triazole), 5.10 (br s, 1H, NH, amine), 4.40 (d, 2H, *J* = 5.9 Hz, CH_2_), 2.26–2.22 (m, 9H, 6H-γ, 3H-β), 1.83–1.74 (m, 6H, H-α), 1.45 (s, 9H, 3 × CH_3_, Boc). ^13^C NMR (CDCl3) *δ* 155.8 (CH, triazole), 79.5 (C, Boc), 59.5 (CH_2_), 42.9 (CH_2_ α), 35.9 (CH_2_ γ), 29.4 (CH_3_, Boc), 28.4 (CH β). The NMR spectra are shown in Figure S1. ESI-MS: *m*/*z* [M + H]^+^ calcd for C_18_H_29_N_4_O_2_: 333.2, found: 333.1.

#### 4.2.2. [1-(Adamantan-1-yl)-1*H*-1,2,3-triazol-4-yl]methylammonium trifluoroacetate (**3**)

Compound **2** (199 mg, 630 μmol) was dissolved in dry dichloromethane (1.9 mL), and TFA (1 mL) was added. The reaction mixture was stirred for 2 h at room temperature. The reaction was monitored via TLC (DCM/MeOH, 3:1). After the reaction was completed, 5% aqueous solution of NaHCO_3_ (25 mL) was added and extracted with DCM (25 mL). The obtained organic layer was washed once more with 5% aqueous solution of sodium bicarbonate, and then dried over anhydrous sodium sulfate. After filtration, the solvent was removed in vacuo, and 126.8 mg (87 %) of compound **3** was obtained as a light brown solid. *R*_f_ = 0.65 (DCM/MeOH, 3:1), mp 98–100 °C. ^1^H NMR (CDCl_3_) *δ* 7.50 (s, 1H, CH, triazole), 3.99 (s, 2H, CH_2_), 2.22–2.25 (m, 9H, 6H-γ, 3H-β), 1.82–1.74 (m, 6H, H-α), 1.62 (s, 2H, NH_2_).^13^C NMR (CDCl_3_) *δ* 117.6 (CH, triazole), 59.5 (CH_2_), 43.0 (CH_2_ α), 35.9 (CH_2_ γ), 29.5 (CH β). The NMR spectra are shown in Figure S2. ESI-MS: *m*/*z* [M]^+^ calcd for C_13_H_21_N_4_: 233.2, found: 233.3.

#### 4.2.3. Benzyl (4*R*)-4-{[1-(adamantan-1-yl)-1*H*-1,2,3-triazol-4-yl]methylaminocarbonyl}-4-[(2S)-2-(*tert*-butyloxycarbonylamino)propanamido]butanoate (**6**)

Dipeptide **5** (139 mg, 340 μmol) was dissolved in dry THF (1 mL), and *N*-methylmorpholine (37.4 μL, 340 μmol) and *n*-butyl chloroformate (43 μL, 340 μmol) were added at −10 °C. After 3 min, compound **3** (102.6 mg, 442 μmol) dissolved in dry DCM (2 mL) was added. The reaction mixture was stirred in an argon atmosphere at −10 °C for 2 h, and an additional 1 h at room temperature after the addition of 5% aqueous solution of sodium bicarbonate. The reaction was monitored via TLC (CHCl_3_/MeOH, 3:1). The aqueous layer was extracted by DCM, and the combined organic extracts were washed with NaHCO_3_(aq) and dried over sodium sulfate. The solvent was removed in vacuo, and the product was purified via column chromatography (CHCl_3_/MeOH, 10:1). A total of 211 mg (95%) of compound **6** was obtained as a white solid. *R*_f_ = 0.85 (CHCl_3_/MeOH, 3:1). ^1^H NMR (CD_3_OD) *δ* 7.89 (br s, 1H, CH, triazole), 7.35–7.29 (m, 5H, CH, Bn), 5.12 (s, 2H, CH_2_, Bn), 4.45 (d, 2H, *J* = 6.9 Hz, CH_2_, linker amide-triazole), 4.38–4.35 (m, 1H, CH, Glu), 4.00 (q, 1H, *J* = 7.1 Hz, CH, Ala), 2.46 (t, 2H, *J* = 7.5 Hz, CH_2_, Glu), 2.23 (s, 9H, 6H-γ, 3H-β), 2.00–1.91 (m, 1H, CH_2_, Glu), 1.86–1.79 (m, 7H, 6H-α, 1H, CH_2_, Glu), 1.35 (s, 9H, 3 × CH_3_, Boc), 1.28 (d, 3H, *J* = 7.1 Hz, CH_3_, Ala). ^13^C NMR (CD_3_OD) *δ* 176.6, 174.2, 173.7 (3 × C=O), 145.7 (C=O, Boc), 137.6 (C, Bn), 129.6, 129.3 (CH, Bn), 120.7 (CH, triazole), 80.8 (C, Boc), 67.6 (CH_2_, Bn), 61.1 (C, triazole), 54.3 (CH, Glu), 52.2 (CH, Ala), 44.0 (CH_2_ α), 37.0 (CH_2_ γ), 36.1 (CH_2_, linker amide-triazole), 31.4 (CH_2_, *iso*Gln), 31.0 (CH β), 28.7 (CH_3_, Boc), 27.8 (CH_2_, Glu), 17.7 (CH_3_, Ala). The NMR spectra are shown in Figure S5. ESI-MS: *m*/*z* [M + H]^+^ calcd for C_33_H_47_N_6_O_6_: 623.4, found: 623.6.

#### 4.2.4. (1*S*)-1-{[*N*-(1*R*)-{3-Benzyloxycarbonyl-1-[1-(adamantan-1-yl)-1*H*-1,2,3-triazol-4-yl]methylaminocarbonyl}propan-1-yl]aminocarbonyl}ethylammonium trifluoroacetate (**7**)

Compound **6** (157.8 mg, 253 μmol) was suspended in dry DCM (1.5 mL), and TFA (0.75 mL) was added. The reaction mixture was stirred for 5 h at room temperature, and the reaction was monitored via TLC (CHCl_3_/MeOH, 3:1). After the reaction was completed, diethyl ether was added, and the solvent was removed in vacuo. The residue was purified via column chromatography (CHCl_3/_MeOH, 5:1), and 130.6 mg (81%) of product **7** was obtained as a white solid. *R*_f_ = 0.69 (CHCl_3_/ MeOH, 3:1). mp 142–144 °C. ^1^H NMR (CD_3_OD) *δ* 7.94 (s, 1H, CH, triazole), 7.37–7.28 (m, 5H, CH, Bn), 5.12 (s, 2H, CH_2_, Bn), 4.44 (s, 2H, CH_2_, linker amid-triazole), 4.40–4.36 (m, 1H, CH, Glu), 3.95 (q, 1H, *J* = 7.0 Hz, CH, Ala), 2.44 (t, 2H, *J* = 7.5 Hz, CH_2_, Glu), 2.23 (br s, 9H, 6H-α, 3H-β), 2.19–2.11 (m, 1H, CH_2_, Glu), 2.03–1.94 (m, 1H, CH_2_, Glu), 1.87–1.79 (m, 6H, H-α), 1.48 (d, 3H, *J* = 7.0 Hz, CH_3_, Ala). ^13^C NMR (CD_3_OD) *δ* 173.3, 171.2 (C=O), 137.5 (C, Bn), 129.6, 129.3, 129.3 (CH, Bn), 120.9 (CH, triazole), 67.6 (CH_2_, Bn), 61.2 (C, triazole), 54.2 (CH, Glu), 50.3 (CH, Ala), 44.0 (CH_2_ α), 36.9 (CH_2_ γ), 35.8 (CH_2_, linker amide-triazole), 31.3 (CH_2_, Glu), 31,0 (CH β), 28,2 (CH_2_, Glu), 17,7 (CH_3_, Ala). The NMR spectra are shown in Figure S6. ESI-MS: *m*/*z* [M]^+^ calcd for C_28_H_39_N_6_O_4_: 523.7, found: 523.6.

#### 4.2.5. (1*S*)-1(4R)-4-{*N*-[1-(Adamantan-1-yl)-1*H*-1,2,3-triazol-4-yl]methylcarbamoyl}-4-[((2*S*)-2-amino)propanamido]butanoic acid (**8**)

Compound **7** (50 mg, 78.5 μmol) was dissolved in ethanol (3 mL), and 10% Pd/C (25 mg) was added. The mixture was stirred for 4 h in a hydrogen atmosphere (*p* = 2.8 bar) at room temperature. The reaction was monitored via TLC (CHCl_3_/MeOH, 3:1). The product was filtered, and the solvent was removed in vacuo. After column chromatography (CHCl_3_/MeOH, 3:1), compound **8** (36,5 mg, 85%, *R*_f_ = 0.45 (CHCl_3_/ MeOH, 3:1)) was obtained as a white solid. Aqueous ammonium formate solution (0.1M) was added next in order to remove the TFA, and the mixture was evaporated in vacuo. Compound **8** was obtained as colorless viscous oil. ^1^H NMR (CD_3_OD) *δ* 7.97 (s, 1H, CH, triazole), 4.47 (d, 2H, *J* = 2.16 Hz, CH_2_, linker amid-triazole), 4.40–4.36 (m, 1H, CH, Glu), 4.00 (q, 1H, *J* = 7.0 Hz, CH, Ala), 2.35 (br s, 2H, CH_2_, Glu), 2.27 (br s, 9H, 6H-α, 3H-β), 2.15–2.14 (m, 1H, CH_2_, Glu), 1.99–1.93 (m, 1H, CH_2_, Glu), 1.90–1.83 (m, 6H, H-α), 1.54 (d, 3H, *J* = 7.0 Hz, CH_3_, Ala). ^13^C NMR (CD_3_OD) *δ* 170.0 (C=O), 119.4 (CH, triazole), 60.4 (C-triazole), 59.8 (C, Ad), 53.2 (CH, Glu), 49.0 (CH, Ala), 42.5 (CH_2_ α), 35.5 (CH_2_ γ), 34.4 (CH_2_, linker amide-triazole), 29.6 (CH β), 27.8 (CH_2_, Glu), 27,3 (CH_2_, Glu), 16.2 (CH_3_, Ala). The NMR spectra are shown in Figure S7. ESI-MS: *m*/*z* [M + H]^+^ calcd for C_21_H_33_N_6_O_4_: 433.3, found: 434.1.

#### 4.2.6. *tert*-Butyl-2-(2,3,4,6-tetra-*O*-benzyl-α-d-mannopyranosyloxy)acetate (**11**)

Compound **10** (249 g, 0.46 mmol) was dissolved in dry *N*,*N*-dimethylformamide (2.5 mL), and then *tert*-butyl bromoacetate (102 μL, 0.69 mmol) and potassium carbonate (318 mg, 2.30 mmol) were added. The reaction mixture was stirred for 48 h at room temperature and subsequently filtered. The beaker was washed with diethyl ether (20 mL). The mixture was extracted three times with water (15 mL). The organic layer was dried over anhydrous sodium sulfate. After filtration, the solvent was removed in vacuo. The product was purified via column chromatography on silica gel (toluene/ EtOAc, 3:1), and compound **11** (188 mg, 62%) was obtained as a yellow viscous oil. *R*_f_ = 0.46 (toluene/ EtOAc, 3:1). ^1^H NMR (CDCl_3_) *δ* 7.40–7.14 (m, 20H, CH, Bn), 5.08 (d, 1H, *J*_1,2_ = 1.7 Hz, H-1), 4.88 (d, 1H, *J*_gem_ = 10.8 Hz, CH_2_, Bn), 4.74 (s, 2H, CH_2_, Bn), 4.65 (d, 1H, *J*_gem_ = 12.1 Hz, CH_2_, Bn), 4.59 (s, 2H, CH_2_, Bn), 4.53 (d, 1H, *J*_gem_ = 12.1 Hz, CH_2_, Bn), 4.49 (d, 1H, *J*_gem_ = 10.8 Hz, CH_2_, Bn), 4.02–3.93 (m, 3H, H-2, H-3, H-4), 4.11 (d, 1H, *J*_gem_ = 16.6 Hz, CH_2_, linker), 3.99 (d, 1H, *J*_gem_ = 16.5 Hz, CH_2_, linker), 3.80–3.69 (m, 3H, H-5, H-6a, H-6b), 1.46 (s, 9 H, *t*-Bu). ^13^C NMR (CDCl_3_) *δ* 169.0 (C=O), 138.5, 138.5, 138.3, 138.3 (4 × C, Bn), 128.3–127.5 (CH, Bn), 97.3 (C1), 79.9, 74.7, 74.5, 72.2 (C2-C5), 75.1, 73.3, 72.6, 72.0 (4 × CH_2_, Bn), 69.2 (C6), 63.5 (CH_2_, linker), 28.1 (CH_3_, Boc). The NMR spectra are shown in Figure S8. ESI-MS: *m*/*z* [M + Na^+^] calcd for C_40_H_46_NaO_8_: 677.3, found: 677.4.

#### 4.2.7. 2-(2,3,4,6-Tetra-*O*-benzyl-α-D-mannopyranosyloxy)acetic acid (**12**)

Compound **11** (177 mg, 0.27 mmol) was dissolved in dry dichloromethane (2 mL), and trifluoroacetic acid (225 μL) was added. The reaction mixture was stirred for 24 h and then diluted with dichloromethane (30 mL). The mixture was washed with water (20 mL), and the water layer was extracted with dichloromethane (20 mL). The organic layers were dried over anhydrous sodium sulfate and, after filtration, the organic solvent was removed in vacuo. The product was purified by column chromatography on silica gel (CHCl_3_/MeOH, 10:1), and compound **12** (123 mg, 76 %) was obtained as a yellow viscous oil. *R*_f_ = 0.19 (CHCl_3_/MeOH, 10:1). ^1^H NMR (CDCl_3_) δ 7.38 -7.13 (m, 20H, CH, Bn), 5.02 (s, 1H, H-1), 4.85 d, 1H, *J*_gem_ = 10.8 Hz, CH_2_, Bn), 4.75 (d, 1H, *J*_gem_ = 12.4 Hz, CH_2_, Bn), 4.72 (d, 1H, *J*_gem_ = 12.4 Hz, CH_2_, Bn), 4,63 (d, 1H, *J*_gem_ = 12.2 Hz, CH_2_, Bn), 4.59 (s, 2H, CH_2_, Bn), 4.53 (d, 1H, *J*_gem_ = 12.2 Hz, CH_2_, Bn), 4.49 (d, 1H, *J*_gem_ = 10.9 Hz, CH_2_, Bn), 4.23 (d, 1H, *J*_gem_ = 17.0 Hz, CH_2_, linker), 4.16 (d, 1H, *J*_gem_ = 17.0 Hz, CH_2_, linker), 3.99–3.90 (m, 3H, H-2, H-3, H-4), 3.80–3.68 (m, 3H, H-5, H-6a, H-6b). ^13^C NMR (CDCl_3_) *δ* 173.8 (C=O), 138.3, 138.2, 138.1, 138.0 (4×C, Bn), 128.3–127.6 (CH, Bn), 97.8 (C1), 79.6, 74.6, 74.3, 72.4 (C2-C5), 75.0, 73.4, 72.7, 72.1 (4 × CH_2_, Bn), 69.1 (C6), 63.2 (CH_2_, linker). The NMR spectra are shown in Figure S9. ESI-MS: *m*/*z* [M + Na^+^] calcd for C_38_H_38_NaO_8_: 621.3, found: 621.2.

#### 4.2.8. Benzyl (4*R*)-4-{*N*-[(1-(adamantan-1-yl)-1*H*-1,2,3-triazol-4-yl]methylcarbamoyl}-4-{(2*S*)-2-[(2,3,4,6-tetra-*O*-benzyl-α-D-mannopyranosyloxy)ethanamido]propanamido}butanoate (**13**)

Compound **12** (96.0 mg, 160 μmol) was dissolved in dry DCM (5 mL). In cooled solution (0 °C), HOBt (21.8 mg, 161 μmol) and EDC × HCl (36.6 mg, 191 μmol) were added. After mixing for 15 min, a solution of derivative **7** (112.3 mg, 176 μmol) in dry 1,4-dioxane (5 mL) and TEA (44.8 μL, 32.5 mg, 321 μmol) was added. The reaction mixture was stirred for 1 h at 0 °C, and then for 24 h at room temperature. The reaction was monitored via TLC (CHCl_3_/MeOH, 9:1). The reaction mixture was diluted with EtOAc (20 mL), and then extracted with 1M HCl and saturated aqueous solution of sodium carbonate._._ The organic layer was dried over sodium sulfate and, after filtration, the solvent was removed in vacuo. The residue was purified via column chromatography (CHCl_3_/MeOH, 9:1), and 176 mg (95%) of product **13** was obtained as a yellow oil. *R*_f_ = 0.75 (CHCl_3_/MeOH, 9:1). ^1^H NMR (CD_3_OD) *δ* 7.82 (s, 1H, triazole), 7.39–7.16 (m, 25H, 25 × CH, Bn), 5.09 (s, 2H, CH_2_, Bn ester), 4.99 (d, 1H, *J*_1,2_ = 1.7 Hz, H-1), 4.69 (s, 2H, CH_2_, linker amide-triazole), 4.60–4.34 (m, 10H, 4 × CH_2_, Bn, CH, Ala, CH, Glu), 4.16–3.90 (m, 5H, CH_2_, acetyl linker, H-2, H-3, H-4), 3.77–3.74 (m, 1H, H-5), 3.71–3.63 (m, 2H, H-6a, H-6b), 2.44 (t, 2H, *J* = 7.3 Hz, CH_2_, Glu), 2.34–2.15 (m, 10H, 6H-γ, 3H-β, 1H, CH_2_, Glu), 2.01–1.90 (m, 1H, CH_2_, Glu), 1.79–1.72 (m, 6H, H-α), 1.35 (d, 3H, *J* = 7.1 Hz, CH_3_, Ala). ^13^C NMR (CD_3_OD) *δ* 175.0, 174.3, 173.6, 171.8 (4 × C=O), 139.9, 139.7 (C, Bn), 129.6–128.7 (CH, Bn), 120.7 (CH, triazole), 99.6 (C1), 81.0, 76.0, 75.8, 73.7 (C2-C5), 75.9, 74.5, 73.8, 73.1 (4 × CH_2_, Bn), 70.4 (C6), 67.6 (CH_2_, Bn ester), 67.2 (CH_2_, acetyl linker), 61.1 (C, triazole), 54.3 (CH, Glu), 50.5 (CH, Ala), 43.9 (CH_2_ α), 36.9 (CH_2_ γ), 36.0 (CH_2_, linker amide-triazole), 31.5 (CH_2_, Glu), 31.0 (CH β), 27.8 (CH_2_, Glu), 18.0 (CH_3_, Ala). The NMR spectra are shown in Figure S10. ESI-MS: *m*/*z* [M + H]^+^ calcd for C_64_H_75_N_6_O_11_: 1103.6, found: 1103.5.

#### 4.2.9. (4*R*)-4-{*N*-[(1-(Adamantan-1-yl)-1*H*-1,2,3-triazol-4-yl]methylcarbamoyl}-4-{(2*S*)-2-[(α-D-mannopyranosyloxy)ethanamido]propanamido}butanoic acid (**14**)

Compound **13** (39 mg, 35.3 μmol) was dissolved in ethanol (3 mL), and 10% Pd/C (27 mg) was added. The mixture was stirred for 48 h in a hydrogen atmosphere (*p* = 2,8 bar) at room temperature. The reaction was monitored via TLC (ACN/H_2_O, 5:1). The reaction mixture was filtered, and the solvent was removed in vacuo. Compound **14** (17 mg, 35%) was obtained as a yellow oil. *R*_f_ = 0.27 (CHCl_3/_MeOH, 3:1). ^1^H NMR (CD_3_OD) *δ* 7.99 (s, 1H, triazole), 4.86 (s, 1H, H-1), 4.51–4.46 (m, 2H, CH_2_, linker amide-triazole), 4.41 (q, 1H, *J* = 7.1 Hz, CH, Ala), 4.34 (dd, 1H, *J* = 9.4 Hz, *J* = 4.4 Hz, CH, Glu), 4.24 (d, 1H, *J*_gem_ = 15.2 Hz, CH_2_, acetyl linker), 4.12 (d, 1H, *J*_gem_ = 15.2 Hz, CH_2_, acetyl linker), 3.97 (dd, 1H, *J*_2,3_ = 3.4 Hz, *J*_1,2_ = 1.7 Hz, H-2), 3.85 (dd, 1H, *J*_6a,6b_ = 11.8 Hz, *J*_5,6a_ = 2.2 Hz, H-6b), 3.80 (dd, 1H, *J*_3,4_ = 9.1 Hz, *J*_2,3_ = 3.4 Hz, H-3), 3.71 (dd, 1H, *J*_6a,6b_ = 11.8 Hz, *J*_5,6b_ = 5.9 Hz, H-6a), 3.64 (app t, 1H, *J* = 9.5 Hz, H-4), 3.59–3.54 (m, 1H, H-5), 2.35 (t, 2H, *J* = 7.3 Hz, CH_2_, Glu), 2.27 (br s, 9H, 6H-γ, 3H-β), 2.22–2.17 (m, 1H, CH_2_, Glu), 2.01–1.92 (m, 1H, CH_2_, Glu), 1.85 (br s, 6H, H-α), 1.42 (d, 3H, *J* = 7.1 Hz, CH_3_, Ala). ^13^C NMR (CD_3_OD) *δ* 175.0, 173.5, 173.4, 170.6 (4 × C=O), 119.4 (CH, triazole), 100.3 (C1), 74.0, 71.0, 70.3, 67.2 (C2-C5), 65.5 (C6), 61.4 (CH_2_, acetyl linker), 59.8 (C, triazole), 56.9 (C, Ad); 52.7 (CH, Glu), 49.1 (CH, Ala), 42.5 (CH_2_ α), 35.5 (CH_2_ γ), 34.4 (CH_2_, linker amide-triazole), 31.5 (CH_2_, Glu), 29.6 (CH β), 27.1 (CH_2_, Glu), 16.6 (CH_3_, Ala). The NMR spectra are shown in Figure S11. ESI-MS: *m*/*z* [M + H]^+^ calcd for C_29_H_45_N_6_O_11_: 653.4, found: 653.4.

### 4.3. Experiments In Vivo

Healthy, nulliparous, and non-pregnant BALB/c female mice 8–12 weeks of age at the initiation of the experiment were used. Mice were obtained from the Ruđer Bošković Institute’s breeding colony. During the experimental period, groups of five animals were kept per cage. The bottoms of the cages were covered with sawdust (Scobis Uno^®^, Mucedola srl, Italy). Standard food for laboratory mice (4RF 21 GLP^®^ Mucedola srl, Italy) was used. Access to food and water was ad libitum. Animals were kept in conventional circumstances: light/dark rhythms of 12/12 h, temperature of 22 °C, and humidity of 55%. All experiments were performed according to the ILAR Guide for the Care and Use of Laboratory Animals, EU Directive 2010/63/EU, and Croatian animal protection law (NN 102/17). Experimental groups of five mice were immunized and boosted twice subcutaneously (s.c.) into the tail base at 21-day intervals. Mice were anesthetized with isoflurane prior to blood collection on the 7th day after the 2nd booster. Sera were collected, decomplemented at 56 °C for 30 min, and stored at –20 °C until tested. The dose of OVA (antigen) was 10 µg per mouse. The doses of PGM and the tested compounds were 200 µg per mouse. OVA and the tested substances were dissolved in water, and the injection volume in all experimental groups was 0.1 mL per mouse.

### 4.4. Enzyme Immunoassays

Enzyme immunoassays (ELISA) were performed on flat-bottomed high-binding microtiter plates (Costar, USA), using murine anti-OVA IgG, IgG1, and IgG2a antibody assay kits (Chondrex, USA), according to the manufacturer’s instructions. The ratio of anti-OVA IgG1 and anti-OVA IgG2a (IgG1/IgG2a) was calculated as an indication of the Th1/Th2 bias of the induced immune response.

### 4.5. Statistics

Statistical analyses were performed using GraphPad Prism Software. Significant differences between experimental groups were evaluated via Kruskal–Wallis ANOVA, followed by Dunn’s multiple comparisons test. Probability values less than 0.05 (*p* < 0.05) were considered significant.

## 5. Conclusions

The immunostimulant activities of adamantyl-triazole DMPs were evaluated and compared to the reference ManAdDMP. In experiments in vivo, adamantyl-triazole DMP **8** exhibited higher adjuvant activity than the reference ManAdDMP. Mannosylation of adamantyl-triazole DMP **8,** which produced the glycoconjugate **14,** resulted in the further amplification of immunostimulant activity. The obtained results indicate the α-COOH of D-Glu as being a suitable position for the attachment of a lipophilic adamantane group, which can also enable a preparation of liposome formulations. Additionally, it was confirmed that the attachment of mannose to the DMPs plays an important role in the stimulation of the immune response. Thus far, glycopeptide **14** has been identified as the most potent adjuvant in the class of mannosylated DMPs. It should also be noted that compound **14** is stable, non-pyrogenic, and water soluble, making it potentially applicable as an adjuvant for vaccines. There is no clear evidence of which PRRs are activated by the mannosylated DMPs, and the possibility of affecting the immune response by binding mannose to mannose receptors cannot be excluded. Therefore, further research on this topic is required.

## Data Availability

The datasets supporting the conclusions of this article are included within the article.

## Supplementary Materials

The following are available online, Figure S1: ^1^H NMR and DEPTQ spectra of compound **2** (CDCl_3_); Figure S2: ^1^H NMR and DEPTQ spectra of compound **3** (CDCl_3_)**; Figure S3**: ^1^H NMR and DEPTQ spectra of compound **4** (D_2_O + TFA); Figure S4: ^1^H NMR and DEPTQ spectra of compound **5** (CDCl_3_); **Figure S5**: ^1^H NMR and DEPTQ spectra of compound **6** (CD_3_OD); Figure S6: ^1^H NMR and DEPTQ spectra of compound **7** (CD_3_OD); Figure S7: ^1^H NMR and DEPTQ spectra of compound **8** (CD_3_OD); Figure S8: ^1^H NMR and DEPTQ spectra of compound **11** (CDCl_3_); Figure S9: ^1^H NMR and DEPTQ spectra of compound **12** (CDCl_3_); Figure S10: ^1^H NMR and DEPTQ spectra of compound **13** (CD_3_OD); Figure S11:^1^H NMR and DEPTQ spectra of compound **14** (CD_3_OD).

## Sample Availability

Samples of the compounds are not available from the authors.
