# Supplementary material for: Design, Synthesis, and Biological Evaluation of Desmuramyl Dipeptides Modified by Adamantyl-1,2,3-triazole
Source: Molecules. 2021 Oct 21;26(21):6352. doi: 10.3390/molecules26216352 (PMC8587862; doi:10.3390/molecules26216352)
Supplement: Supplementary file 1 [file molecules-26-06352-s001.zip › molecules-1425010-supplementary.pdf]

Supporting Information

for

Design, synthesis and biological evaluation of desmuramyl dipeptides modified by adamantly-1,2,3-triazole

Vesna Petrović Peroković, Željka Car, Josip Draženović, Ranko Stojković, Lidija Milković, Mariastefania Antica, Đani Škalamera, Srđanka Tomić and Rosana Ribić

*

1. Experimental and characterization data

1.1. Preparation of azidoadamantane (**1**)

Commercially available adamantan-1-ol (500.4 mg, 3.29 mmol) was dissolved in a mixture of chloroform (7 mL) and 57% sulfuric acid solution (7 mL). The prepared mixture was cooled to 0 ° C and sodium azide (426.9 mg, 6.57 mmol) was added in small portions over half an hour. The reaction mixture was stirred at room temperature for 3 hours then poured into ice-cold water (20 mL). The prepared mixture was extracted with DCM (4×50 mL), and the combined organic layers were washed with aqueous 5% $NaHCO_3$ (aq) (50 mL) and water (50 mL). Organic layers were dried over sodium sulfate. The dried organic extract was evaporated on a rotary evaporator. 510.0 mg (87%) of compound **1** was obtained as a white solid. ^{1}H NMR (DMSO-d_6) δ 2.10 (s, 3H, H-β), 1.76 (d, J = 3.06 Hz, 6H, H-α), 1.64 (s, 6H, H-γ). ^{13}C NMR (DMSO-d_6) δ: 58.9 (C); 40.8 (CH_2 α); 35.1 (CH_2 γ); 29.1 (CH β).

1.2. Preparation of D-Glu(OBn)OH (**4**)

D-Glu-OH (2.02 g, 13.7 mmol) was suspended in freshly distilled benzyl alcohol (15 mL) and BF_3×Et_2O (7×0.5 mL) was added dropwise to the suspension thus prepared over one hour and under an argon atmosphere. The reaction was stirred under argon for 20 hours after which time THF (45 mL) and TEA (3.8 mL) were added to the reaction mixture to give a milky white mixture. The solvent was then evaporated to dryness on a rotary evaporator, and hexane: EtOAc = 3: 2 (10 mL) was added to the evaporated residue. The resulting mixture was stirred and filtered through a sintered funnel (pore 3). The residual precipitate was washed with hexane/ EtOAc, 3:1 (10 mL) and air dried. The compound was recrystallized from H_2O/EtOH, 9:1 (50 mL) and filtered off under reduced pressure. 2.00 g (61%) of product **4** were obtained as a white solid. R_f = 0.18 ($CHCl_3$/MeOH =3:1); mp 157.5-159 °C. ^{1}H NMR (D_2O + TFA) δ 7.38-7.32 (m, 5H, CH, Bn); 5.10 (s, 2H, CH_2, Bn); 4.04 (t, 1H, J = 6.8 Hz, CH, Glu); 2.65-2.58 (m, 2H, CH_2, Glu); 2.24-2.12 (m, 2H, CH_2, Glu). ^{13}C NMR (D_2O + TFA) δ 174.0, 171.3 (2×C=O); 135.3 (C, Bn); 128.8, 128.6, 128.3 (CH, Bn); 67.2 (CH_2, Bn); 51.9 (CH, Glu); 29.5 (CH_2, Glu); 24.7 (CH_2, Glu). ESI-MS: *m/z* $[M+Na]^+$ calcd for $C_{12}H_{15}NNaO_4$ 260.1, found: 260.2.

1.3. Preparation of Boc-L-Ala-D-Glu(OBn)-OH (**5**)

Compound **4** (203 mg, 856 μmol) was dissolved in dry tetrahydrofuran (6 mL), and TEA (2 × 114 μL, 1.636 mmol over 5 min) was added to the thus prepared solution cooled to 0 ° C under argon) and Boc-L-Ala-OSu (238 mg, 831 μmol). The prepared mixture was continued to stir for an additional 30 min in an ice bath and then for 24 h at room temperature under an argon

atmosphere. The reaction was monitored by TLC ($CHCl_3$/MeOH, 3: 1). After 24 h, the reaction mixture was evaporated to dryness and the evaporated residue was dissolved in ethyl acetate (20 mL). The resulting solution was washed with 1M HCl (20 mL), and the aqueous layer was extracted with ethyl acetate (2×20 mL). The combined organic layers were washed with saturated NaCl solution (2×30 mL), dried over sodium sulfate and evaporated on a rotary evaporator. Product **5** (331 mg, 95%) was obtained as a colorless glassy solid. R_f = 0.33 ($CHCl_3$/MeOH, 3:1), mp = 77-78 ° C. ^{1}H NMR ($CDCl_3$) δ 7.34-7.31 (m, 5H, CH, Bn); 5.11 (s, 2H, CH_2, Bn); 4.62 (br s, 1H, CH, Glu); 4.38-4.30 (m, 1H, CH, Ala); 2.56-2.39 (m, 2H, CH_2, Glu); 2.32-2.23 (m, 1H, CH_2, Glu); 2.09-2.00 (m, 1H, CH_2, Glu); 1.41 (s, 9H, 3×CH_3, Boc); 1.33 (d, 3H, J = 5.0 Hz, CH_3, Ala). ^{13}C NMR ($CDCl_3$) δ 176.5, 174.2, 173.4, 172.8 (4×C=O); 135.7 (C, Bn); 128.5, 128.3 (CH, Bn); 80.7 (C, Boc); 66.6 (CH_2, Bn); 51.5 (CH, Glu); 49.8 (CH, Ala); 30.2 (CH_2, Glu); 28.3 (CH_3, Boc); 27.2 (CH_2, Glu); 18.3 (CH_3, Ala). ESI-MS: *m/z* $[M+Na]^+$ calcd for $C_{20}H_{28}N_2NaO_7$: 431.2, found: 431.2.

1.4. Preparation of methyl 2,3,4,6-tetra-*O*-benzyl α-D-mannopyranoside **(9)**

Methyl α-D-mannopyranoside (5.0 g, 0.026 mol) was dissolved in dry dioxane (50 mL). The solution was stirred on a magnetic stirrer at room temperature and potassium hydroxide (25.3 g, 0.451 mol) was added gradually. Benzyl bromide (26 mL, 0.219 mol) was added dropwise to the reaction mixture over 1 hour, then the whole mixture was heated to 100 ° C and refluxed for 24 h. The reaction was monitored by TLC (toluene/EtOAc, 10:1) by spraying with 10% sulfuric acid with heating. After completion of the reaction, water (50 mL) was added to the flask and extracted with diethyl ether (2×50 mL), and the combined ether extracts were washed with water (50 mL) and evaporated on a rotary evaporator. Product **9** (8.7 g , 61%) was obtained in the form of a yellow oily liquid. R_f = 0.50 (toluene/EtOAc,10: 1), ^{1}H NMR ($CDCl_3$) δ 7.38-7.19 (m, 20H, CH, Bn), 4.81 (s, 1H, H-1), 4.79-4.47 (m, 8H, 4×CH_2, Bn), 3.88 (br s, 1H, H-2), 3.77 (app t, 1H, J = 9.5 Hz, H-4), 3.73 (dd, 1H, $J_{2,3}$ = 2.8 Hz, $J_{3,4}$ = 9.3 Hz, H-3), 3.68-3.63 (m, 2H, H-6a, H-6b), 3.57-3.54 (m, 1H, H-5), 3.27 (s, 3H, OCH_3). ^{13}C NMR ($CDCl_3$) δ 138.4, 138.2, 138.3, 138.2 (4C, Ar), 128.3-127.3 (CH, Ar), 98.9 (C1), 80.2, 74.9, 74.6, 71.6 (C2-C5), 74.9, 73.3, 72.5, 72.0 (4 CH_2, Bn), 69.3 (C6), 54.6 (OCH_3).

1.5. Preparation of 2,3,4,6-tetra-*O*-benzyl-α-D-mannopyranose (**10**)

Glacial acetic acid (24.9 mL) and 3M sulfuric acid (7.8 mL) were added to methyl 2,3,4,6-tetra-*O*-benzyl-α-D-mannopyranoside **9** (5.0 g, 9.01 mmol). The solution was stirred for 5.5 hours at 90 °C. The course of the reaction was monitored by TLC (toluene/EtOAc, 5:1) with spraying with 10% sulfuric acid and heating. After completion of the reaction, saturated NaCl solution (30 mL) was added to the separatory funnel, and the reaction mixture was transferred to a funnel and extracted with diethyl ether (2×30 mL). The ether extracts were dried over anhydrous Na_2SO_4, filtered, and evaporated to dryness on a rotary evaporator. The product was purified by column chromatography on silica gel (toluene/EtOAc, 5:1). 1.46 g (30%) of pure product **10** was obtained as a yellow, oily liquid. $R_f = 0.34$ (toluene/EtOAc, 5:1). ^{1}H NMR ($CDCl_3$) δ 7.38-7.27 (m, 20H, CH, Bn), 4.94 (d, 1H, $J_{gem} = 11.5$ Hz, CH_2, Bn), 4.86-4.66 (m, 4H, 2×CH_2, Bn), 4.68 (d, 1H, $J_{1,2} = 3.6$ Hz, H-1), 4.57 (d, 1H, $J_{gem} = 11.5$ Hz, CH_2, Bn), 4.49-4.37 (m, 2H, CH_2, Bn), 4.06-4.01 (m, 1H, H-2), 3.94-3.87 (m, 3H, H-3, H-4, H-5), 3.60-3.51 (m, 2H, H-6a, H-6b). ^{13}C NMR ($CDCl_3$) δ 138.8, 138.6, 138.5, 137.9 (4C, Bn), 128.4-127.3 (CH, Bn), 98.7 (C1), 79.1, 76.4, 75.1, 69.2 (C2-C5), 74.7, 73.5, 73.4, 73.2 (4 CH_2, Bn), 69.0 (C6).

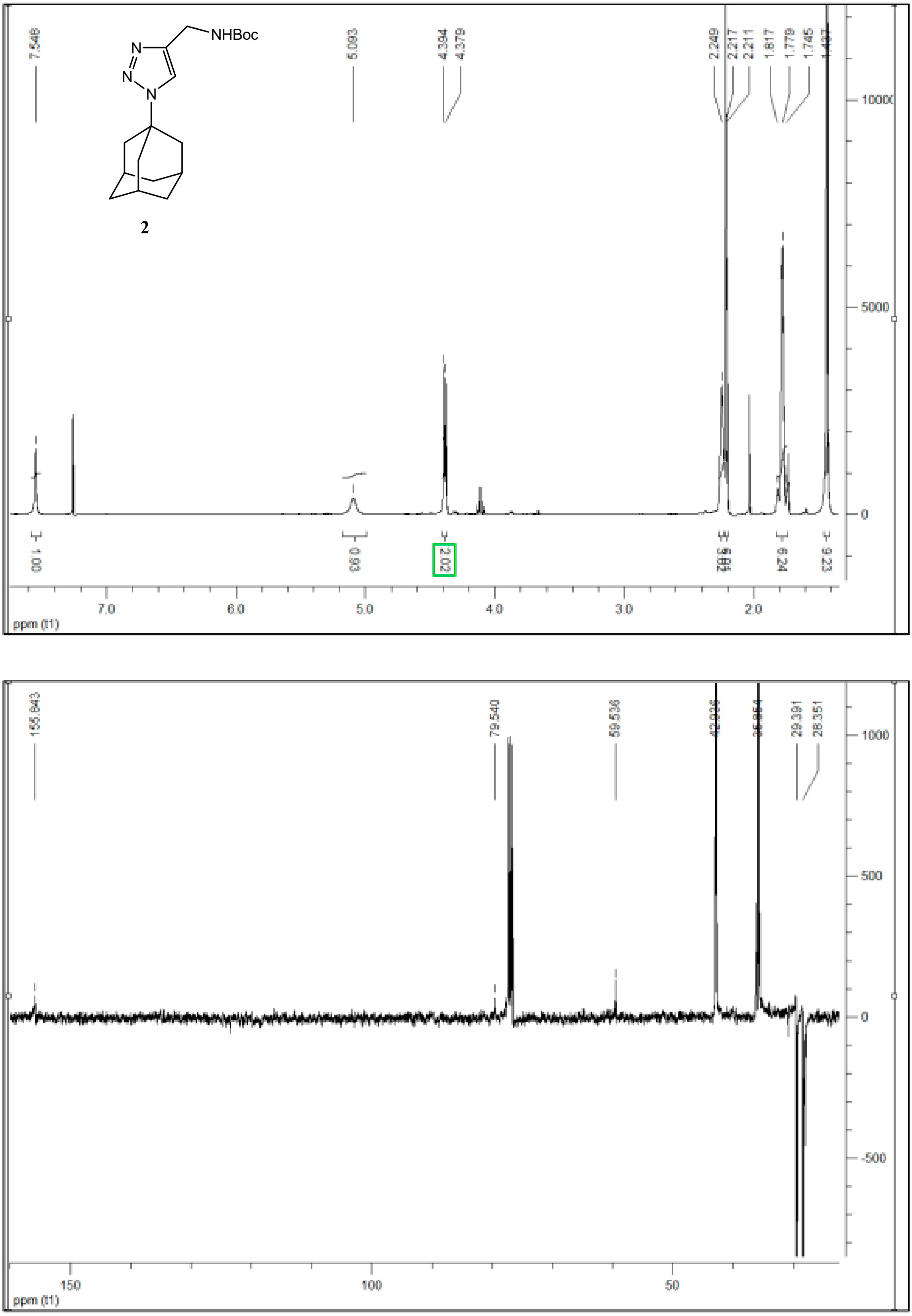

Figure S1. ^{1}H NMR and DEPTQ spectra of compound **2** ($CDCl_3$)

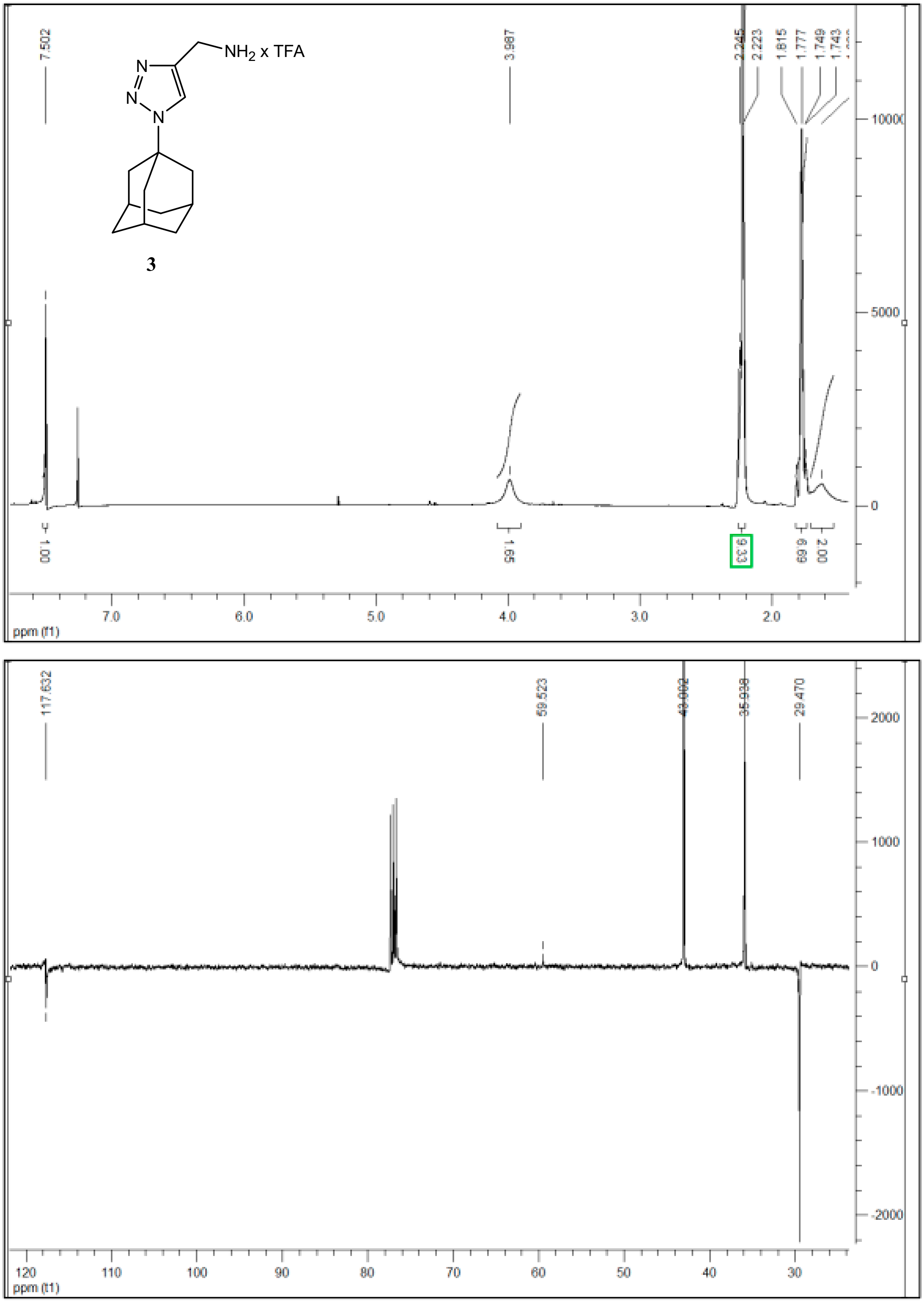

Figure S2. ^{1}H NMR and DEPTQ spectra of compound **3** ($CDCl_3$)

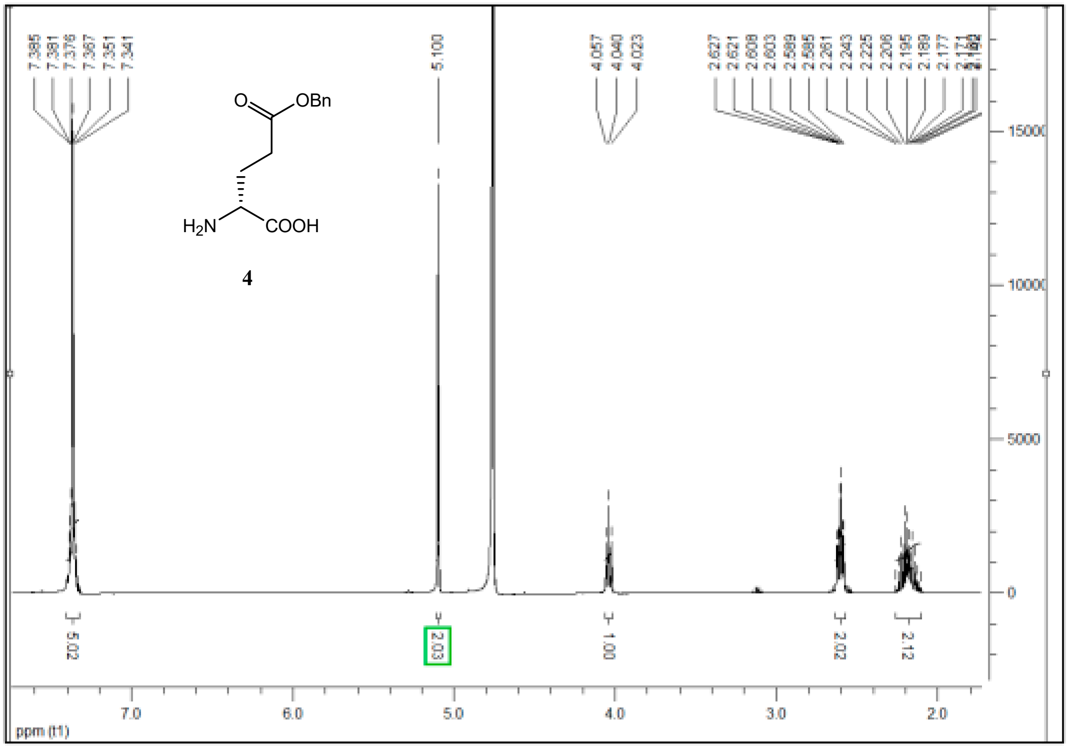

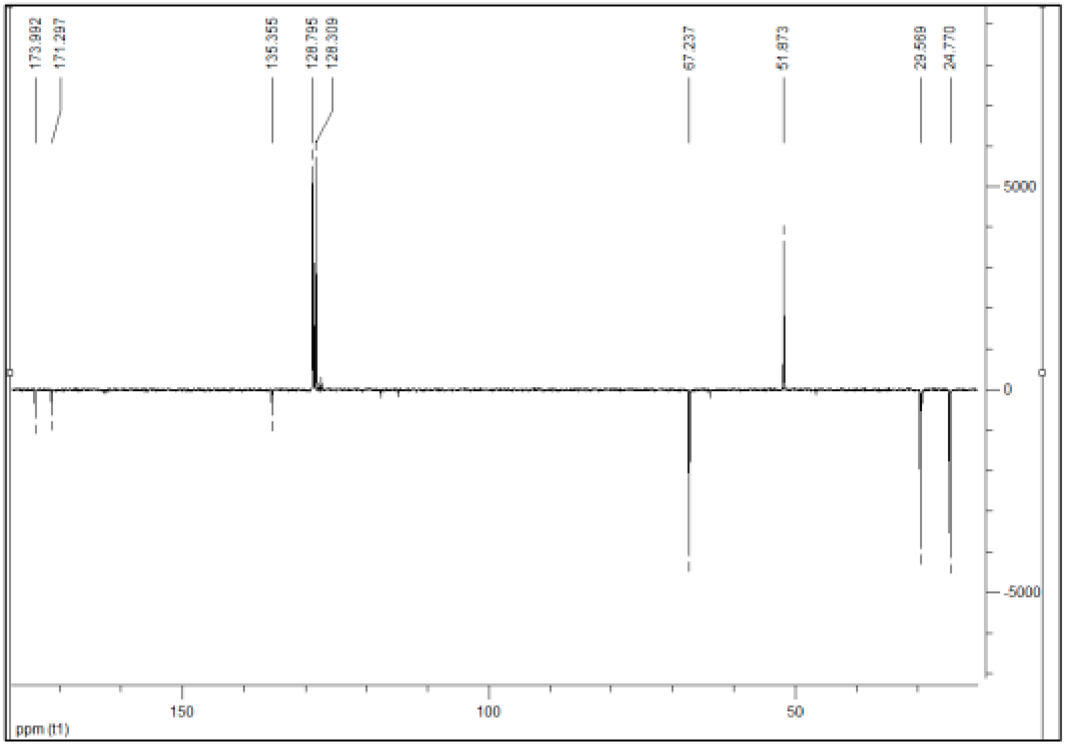

Figure S3. ^{1}H NMR and DEPTQ spectra of compound **4** (D_2O + TFA)

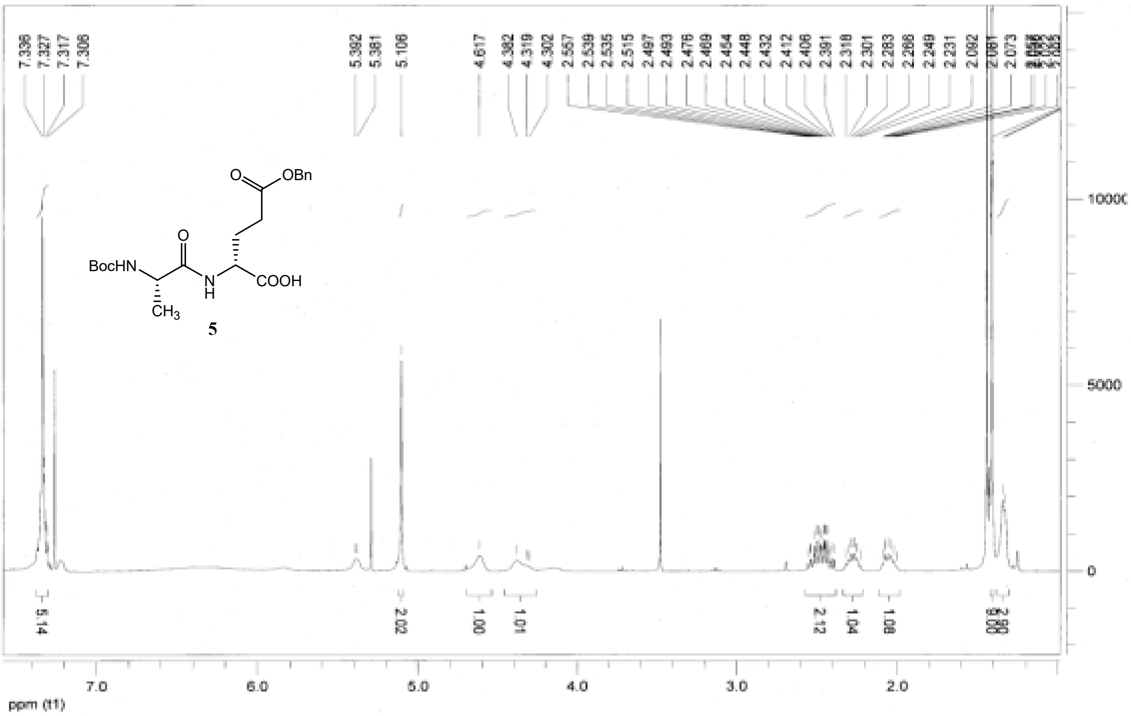

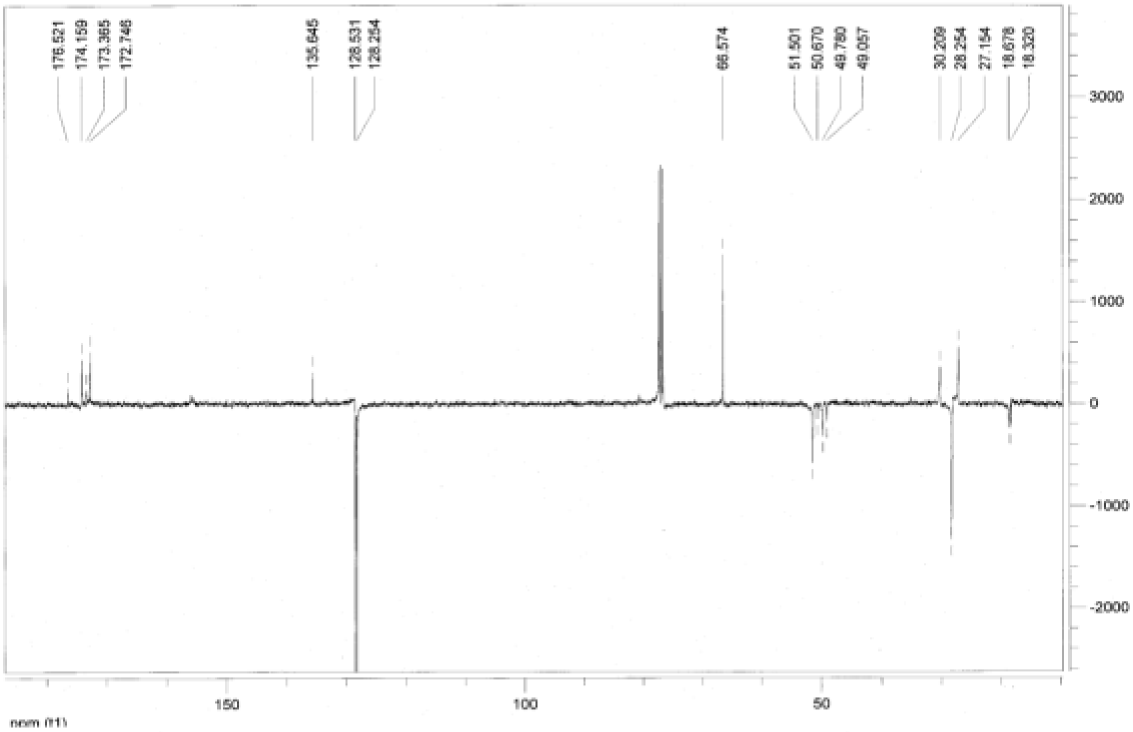

Figure S4. ^{1}H NMR and DEPTQ spectra of compound **5** ($CDCl_3$)

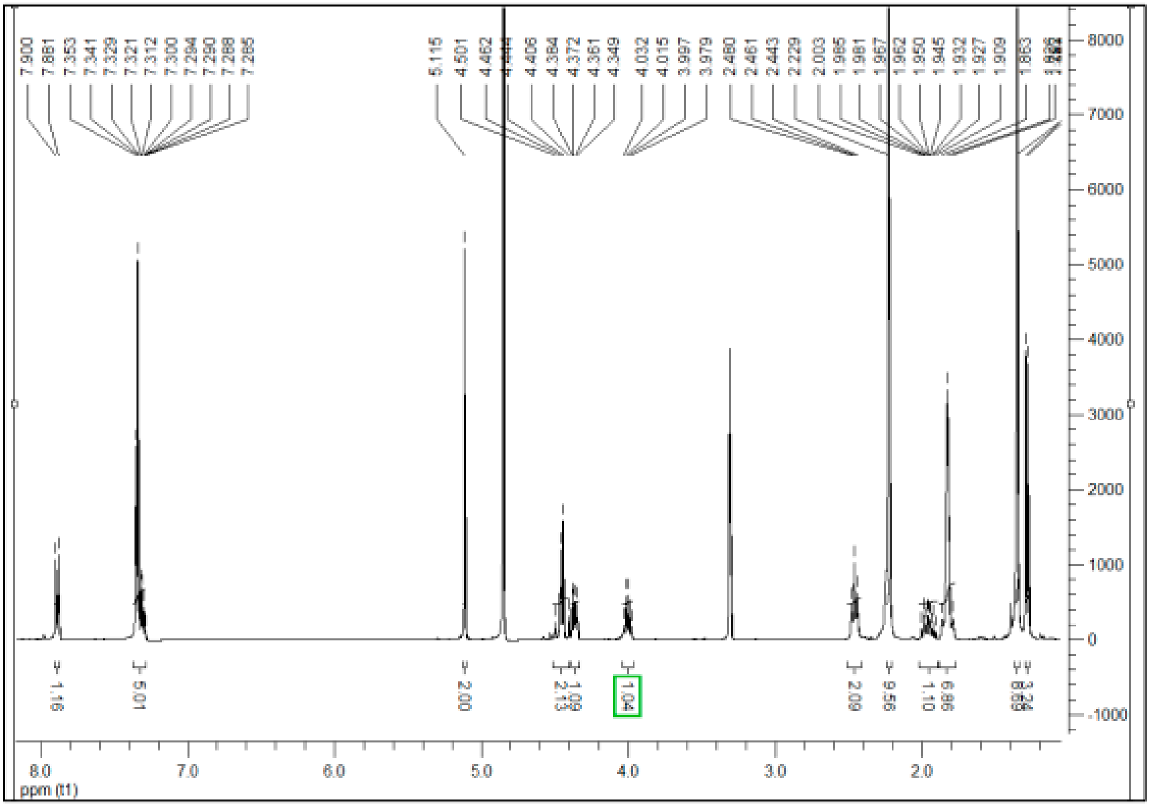

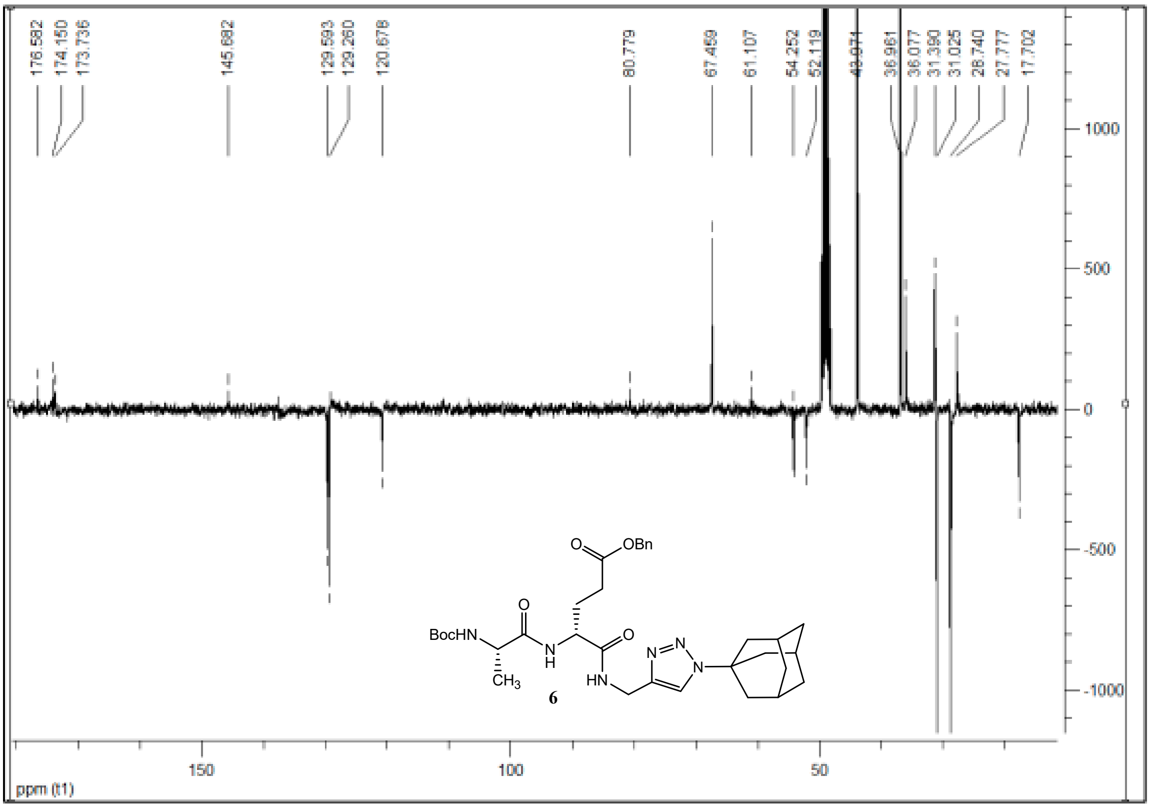

Figure S5. ^{1}H NMR and DEPTQ spectra of compound **6** (CD_3OD)

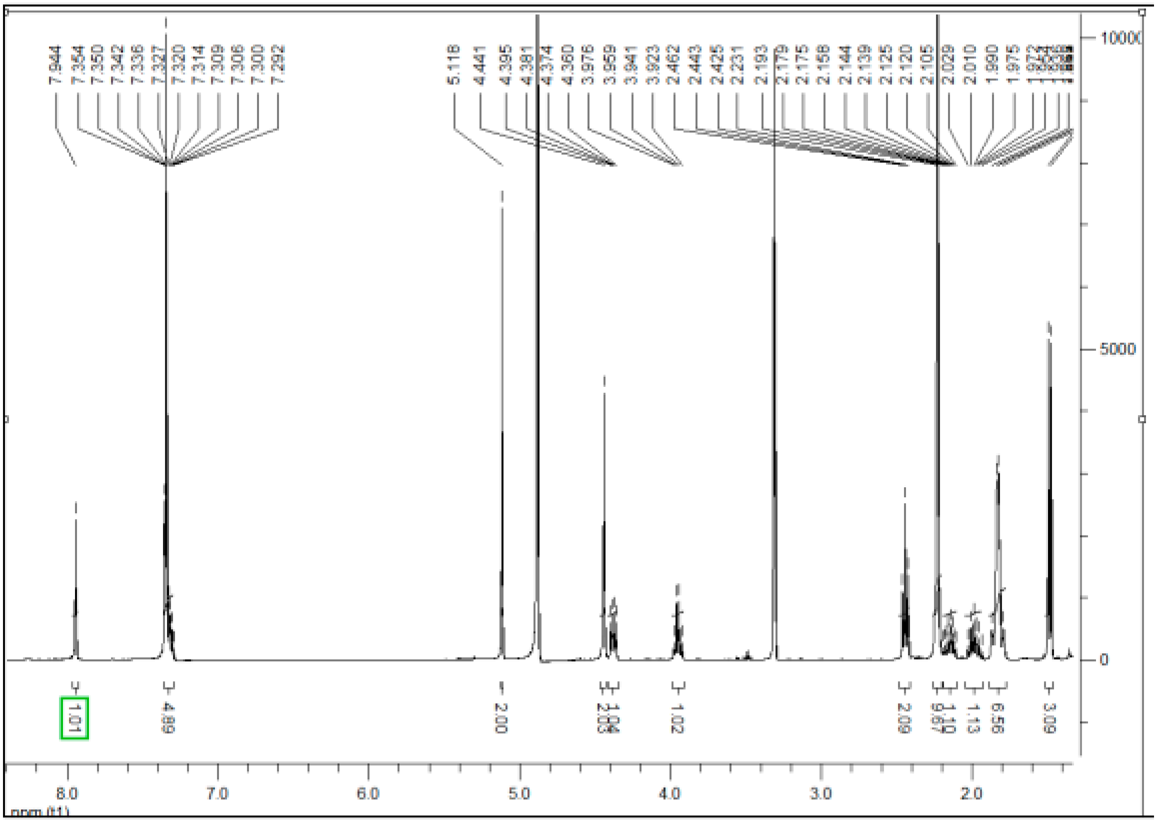

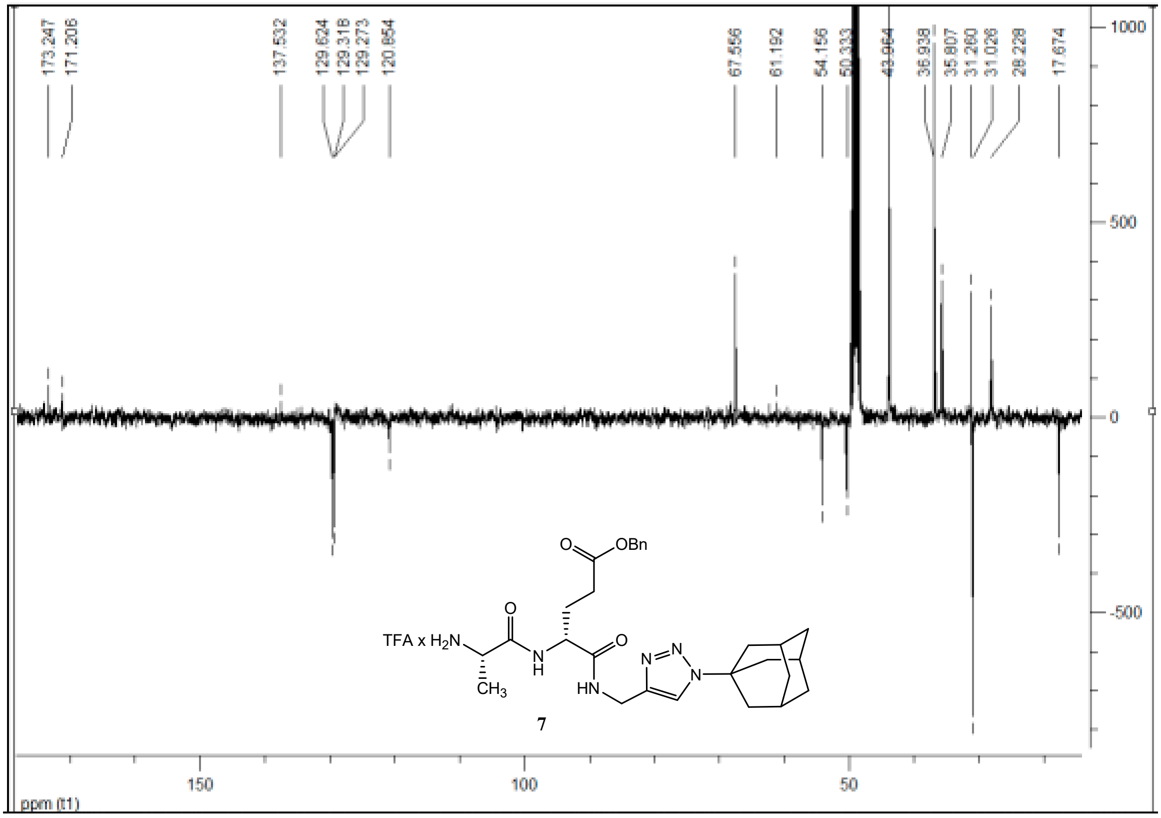

Figure S6. ^{1}H NMR and DEPTQ spectra of compound **7** (CD_3OD)

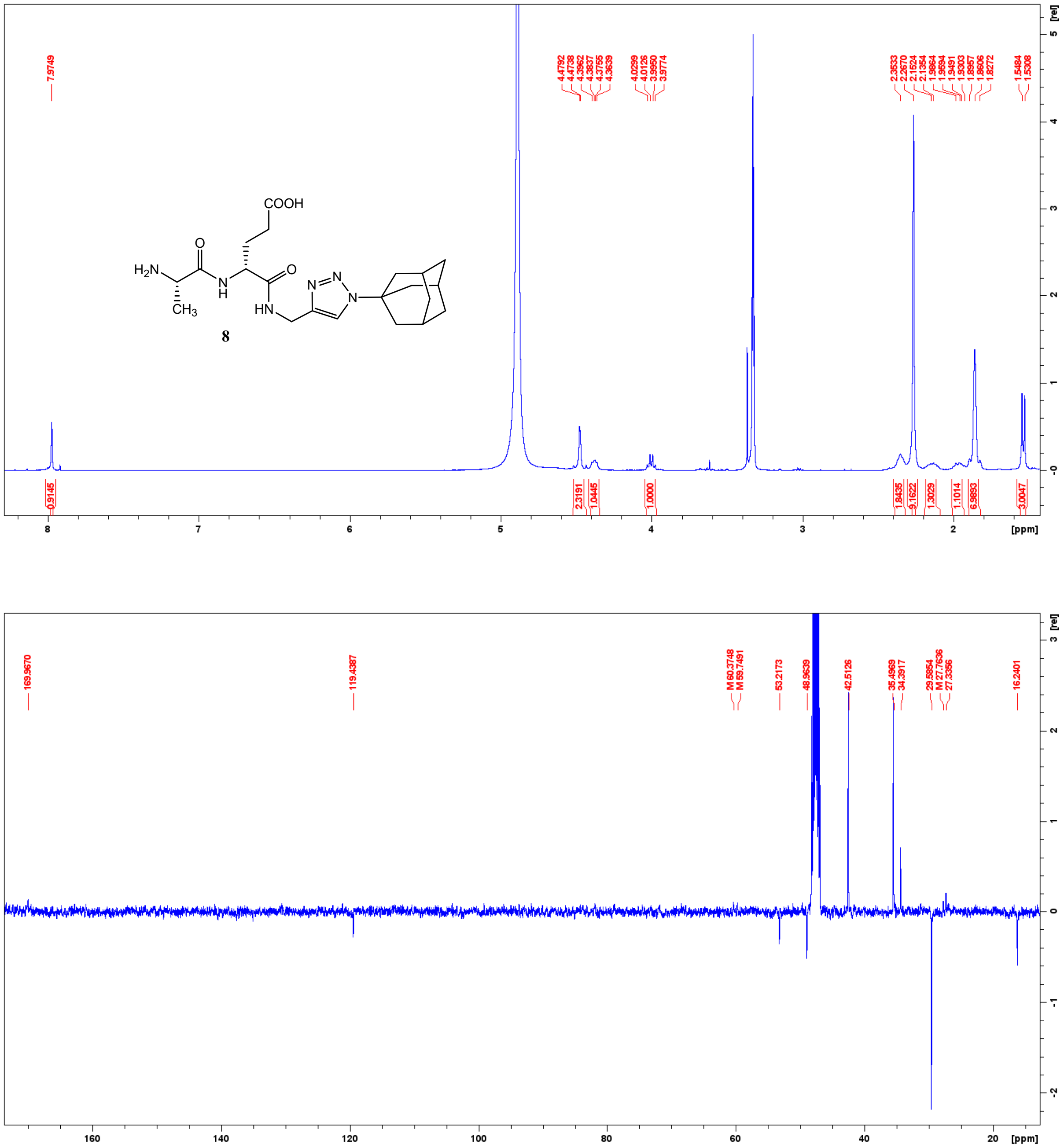

Figure S7. ^{1}H NMR and DEPTQ spectra of compound **8** (CD_3OD)

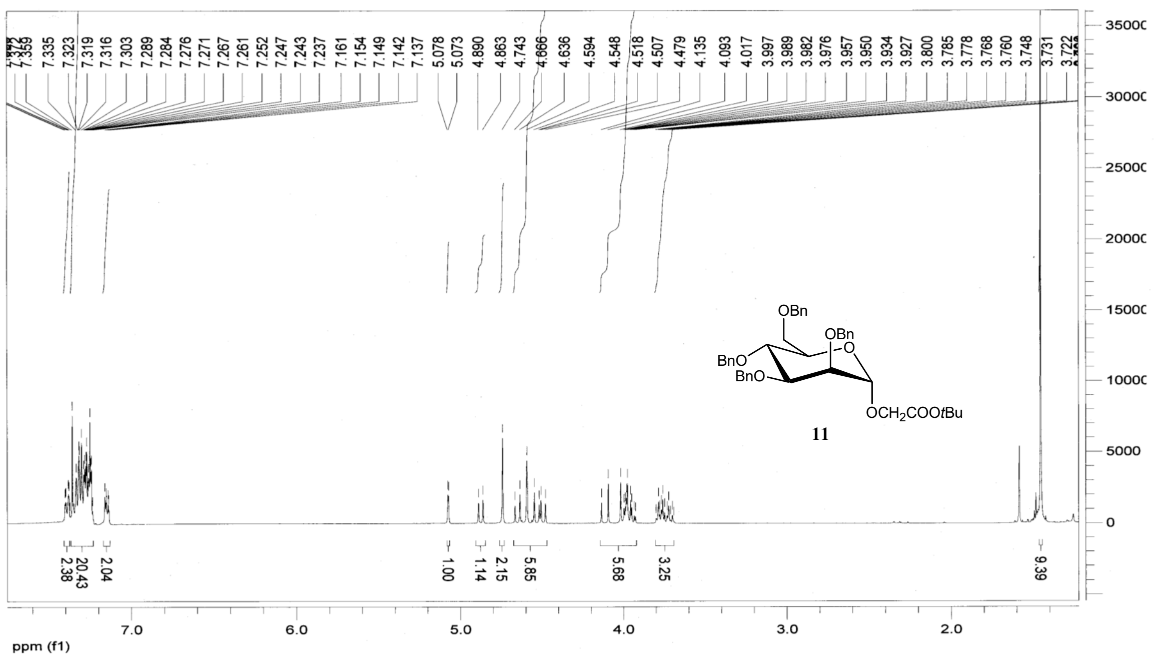

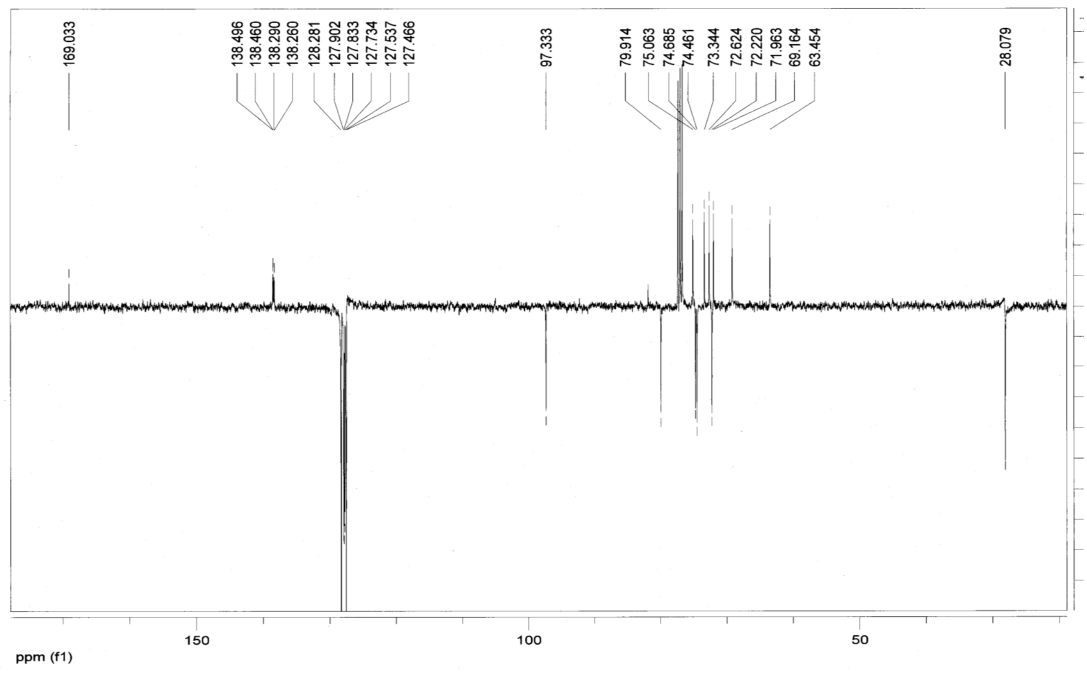

Figure S8. ^{1}H NMR and DEPTQ spectra of compound **11** ($CDCl_3$)

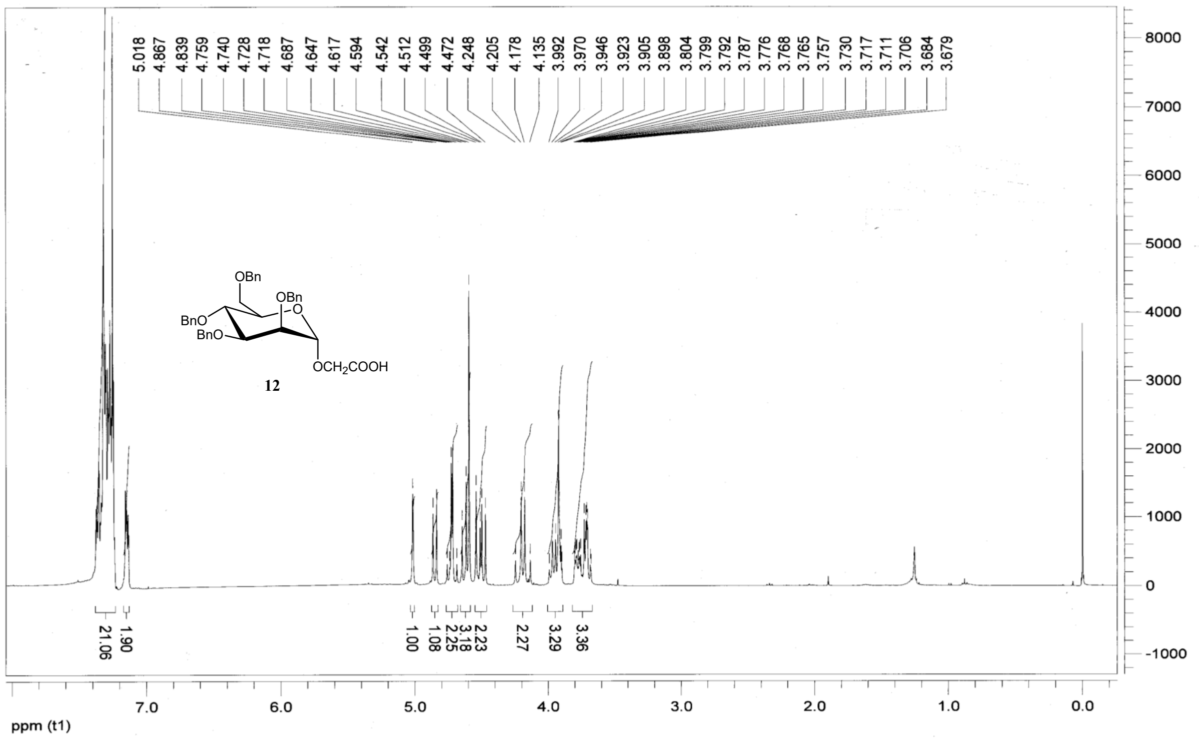

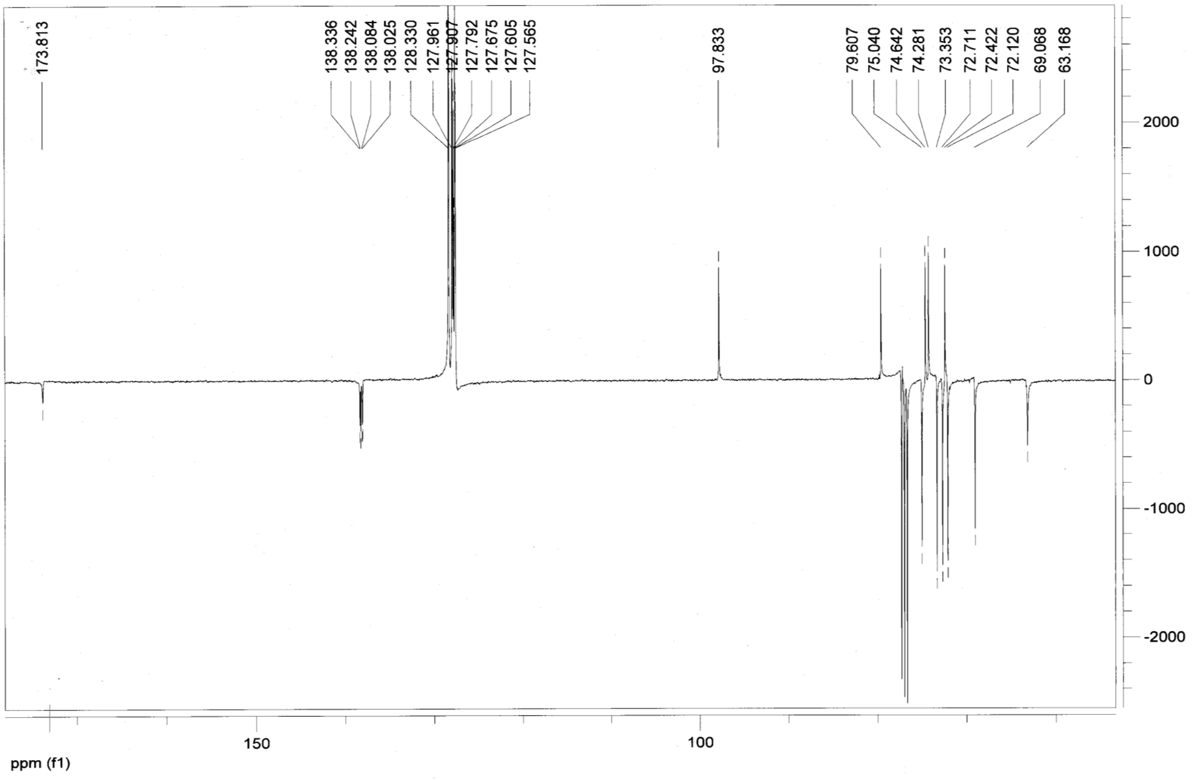

Figure S9. ^{1}H NMR and DEPTQ spectra of compound **12** ($CDCl_3$)

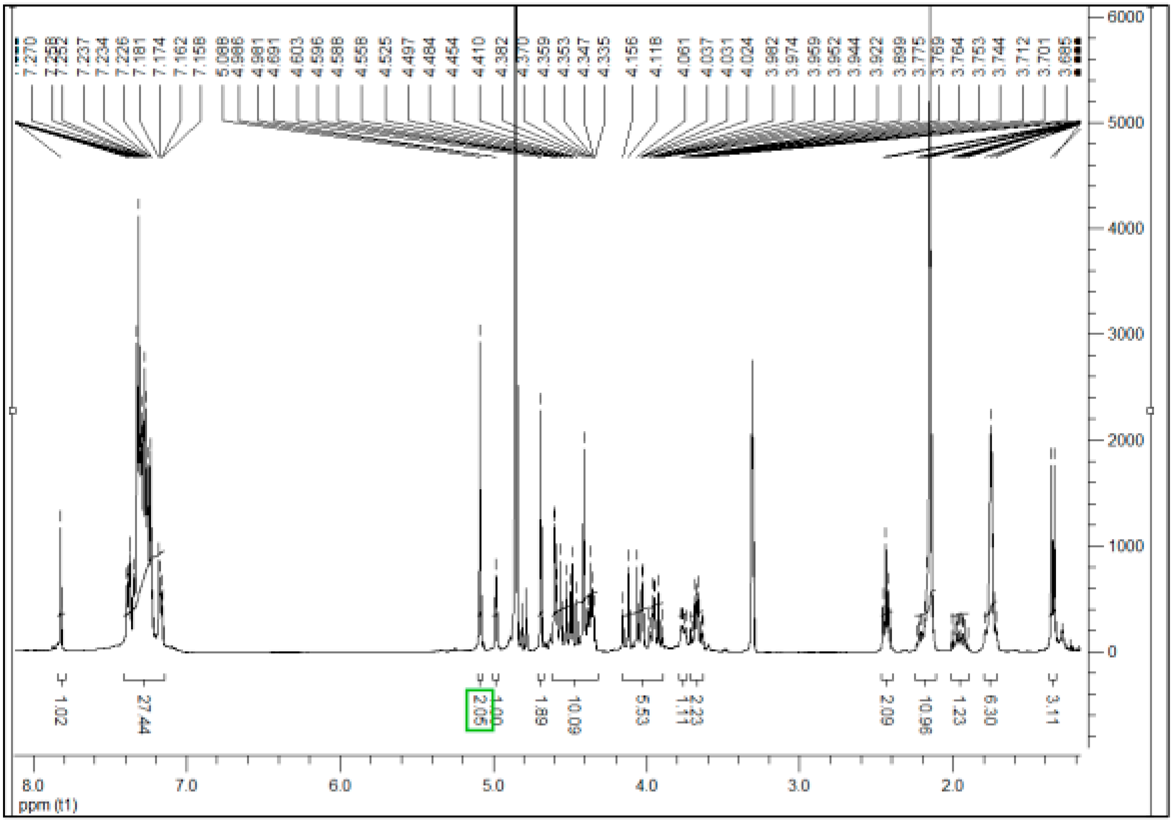

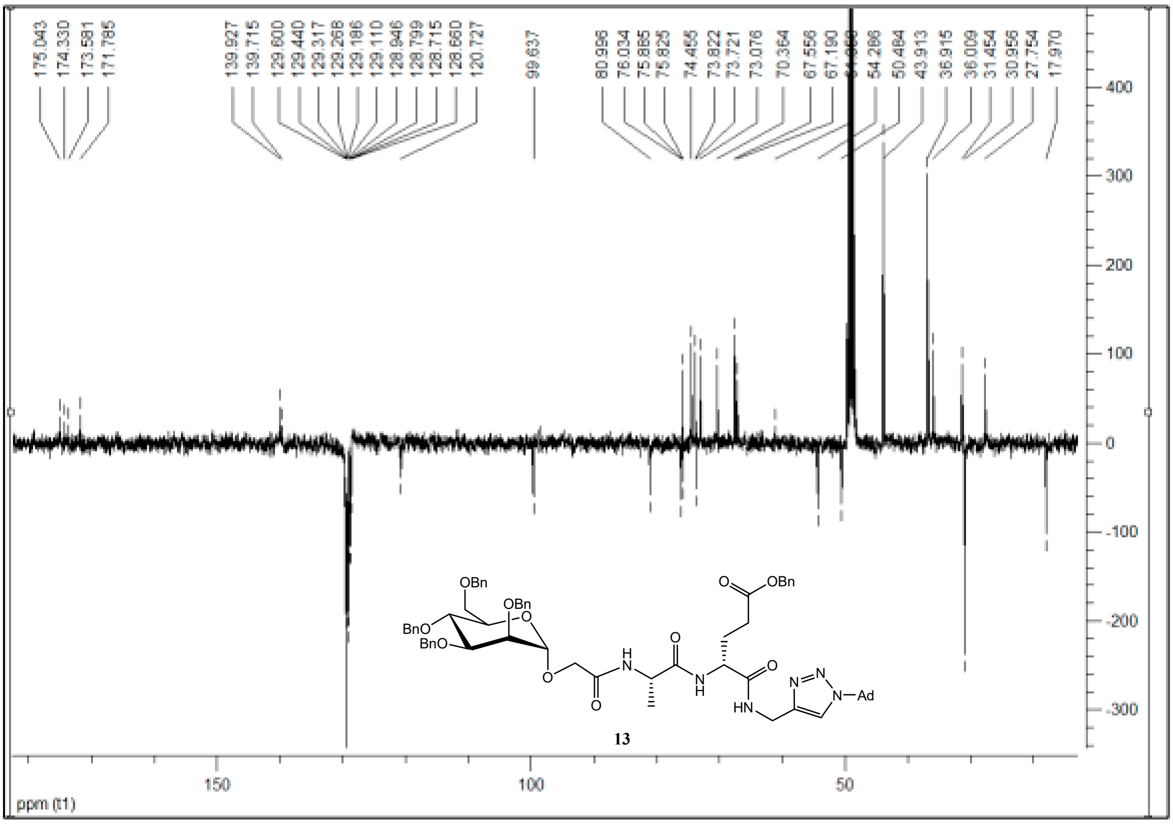

Figure S10. ^{1}H NMR and DEPTQ spectra of compound **13** (CD_3OD)

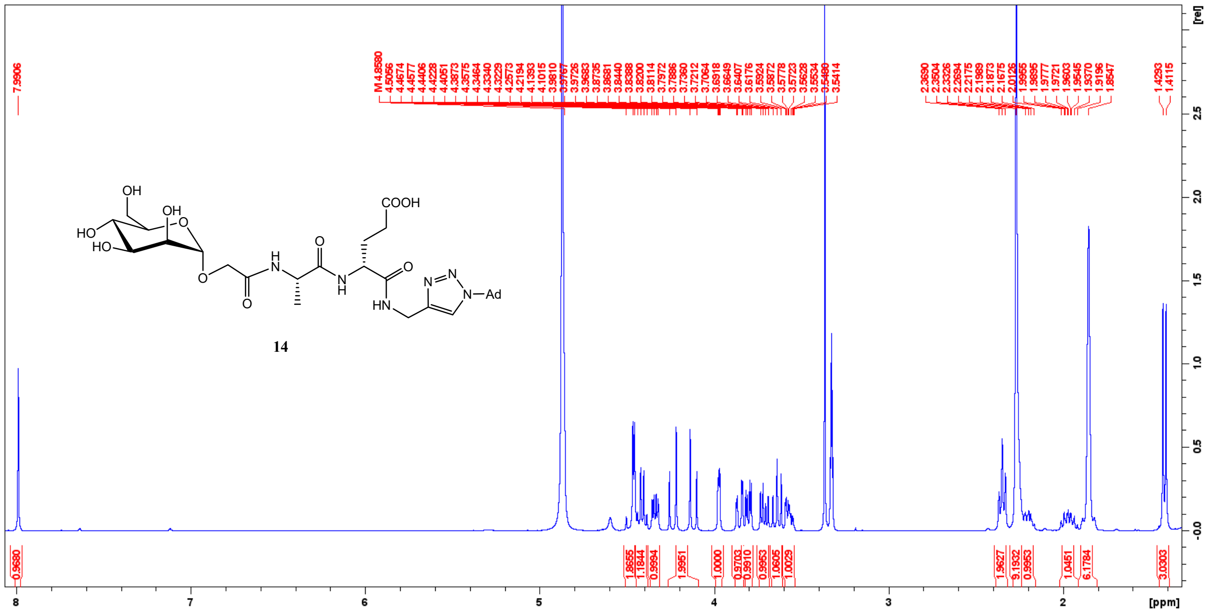

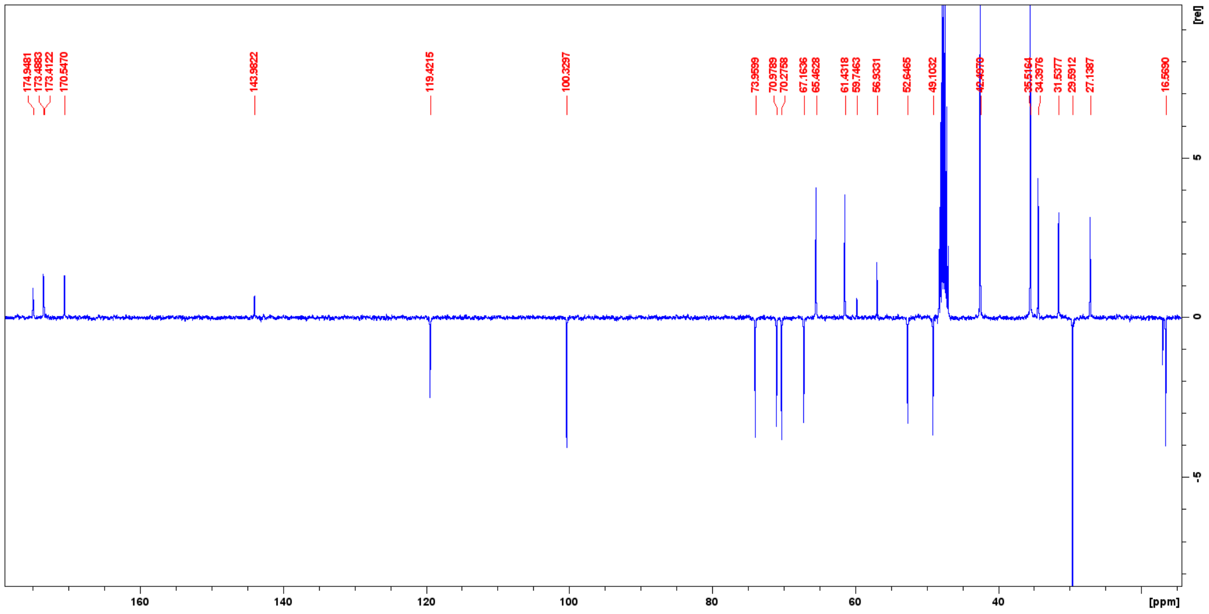

Figure S11. ^{1}H NMR and DEPTQ spectra of compound **14** (CD_3OD)
